# Exploring the α-Glucosidase Inhibitory Activity of Bioactive Compounds from *Persicaria odorata* (Lour.) Extracts via Integrated In Vitro and In Silico Studies

**DOI:** 10.3390/ijms27156885

**Published:** 2026-08-01

**Authors:** Muhammad Subhan, Kamonpan Sanachai, Bodee Nutho, Juthamat Ratha, Pimolwan Siriparu, Suthida Datham, Benjamat Bangthong, Tanit Padumanonda, Bunleu Sungthong, Ploenthip Puthongking

**Affiliations:** 1 Graduate School in Pharmaceutical Chemistry and Natural Products, Faculty of Pharmaceutical Sciences, Khon Kaen University, Khon Kaen 40002, Thailand; muhammad.s@kkumail.com; 2 Department of Biochemistry, Faculty of Sciences, Khon Kaen University, Khon Kaen 40002, Thailand; kamosa@kku.ac.th; 3 Department of Pharmacology, Faculty of Science, Mahidol University, Bangkok 10400, Thailand; bodee.nut@mahidol.ac.th; 4 Melatonin Research Group, Khon Kaen University, Khon Kaen 40002, Thailand; juthra@kku.ac.th (J.R.); pimolwan.s@kkumail.com (P.S.); suthida.tham@kkumail.com (S.D.); benjamat.b@kkumail.com (B.B.); 5 Division of Pharmacology and Toxicology, Faculty of Pharmaceutical Sciences, Khon Kaen University, Khon Kaen 40002, Thailand; tanpad@kku.ac.th; 6 Integrative Pharmaceuticals and Innovation of Pharmaceutical Technology Research Unit, Faculty of Pharmacy, Mahasarakham University, Maha Sarakham 44150, Thailand; bunleu.s@msu.ac.th; 7 Division of Pharmaceutical Chemistry, Faculty of Pharmaceutical Sciences, Khon Kaen University, Khon Kaen 40002, Thailand

**Keywords:** type 2 diabetes mellitus, phenolics, rutin, procyanidin B, molecular docking, molecular dynamics

## Abstract

*Persicaria odorata* (Lour.) contains considerable amounts of phytochemicals, including phenolics and essential oils, which have been reported to inhibit α-glucosidase activity. However, which bioactive compounds play a key role in inhibiting the α-glucosidase activity is unclear. To address this issue, the methanolic extract of *P. odorata* was assayed via in vitro studies, and forty-four compounds in *P. odorata* extracts were elucidated using in silico studies. The extract strongly inhibited yeast α-glucosidase with an IC_50_ value of 0.31 µg/mL, which was 450-fold more potent than acarbose (IC_50_ = 139.47 µg/mL), under experimental conditions. The Lineweaver–Burk plots indicated that the inhibition type of the extract on α-glucosidase is a noncompetitive inhibitor. Molecular docking demonstrated that procyanidin B (**P9**) and rutin (**P19**), which belong to the flavonoids, were predicted by computational approaches to be bioactive compounds that have more favorable binding interactions to yeast α-glucosidase residues compared to forty-four other compounds and acarbose. Molecular dynamics simulations revealed that **P9** and **P19** complexes with yeast α-glucosidase remained stable during the 500 ns simulations. Moreover, both **P9** and **P19** were predicted to qualify as α-glucosidase inhibitors with low toxicity. These findings suggest that *P. odorata* could be used as an alternative edible plant to manage postprandial hyperglycemia in T2DM, and could serve as basic scientific data. Further experimental studies of the pure bioactive compounds are necessary to support these findings.

## 1. Introduction

Type 2 diabetes mellitus (T2DM), one of the most prevalent chronic metabolic disorders characterized by hyperglycemia, arises when the body is unable to effectively utilize insulin or when the pancreas releases insufficient insulin levels [1]. Over 90% of diabetic patients are diagnosed with type 2 diabetes (T2D), which is non-insulin-dependent diabetes mellitus [2]. Hyperglycemia in diabetic patients could elevate the risk of vascular complications like retinopathy, nephropathy, and neuropathy [3], and also have the potential to directly elevate the risk of other deadly diseases, such as cardiovascular diseases, stroke, and cancer [2]. Controlling and suppressing postprandial hyperglycemia by inhibiting α-glucosidase activity has been implemented as one of the most effective strategies for both preventing and treating T2D [4].

α-Glucosidase, situated in the brush border of the small intestine, is a critical hydrolytic enzyme that is essential for the digestion of carbohydrates. It is responsible for the regulation of blood glucose levels by specifically hydrolyzing the 1→4-α-glucopyranosidic bond to produce α-D-glucose [5,6]. α-Glucosidase inhibitor agents (acarbose, voglibose, and miglitol) have been known to act as competitive inhibitors of the α-glucosidases [3], which can reduce both postprandial hyperglycemia and hyperinsulinemia, thereby enhancing insulin sensitivity and alleviating the stress on β-cells [3]. However, their undesired effects on the gastrointestinal tract have limited their efficacy, such as flatulence and diarrhea, which are linked to the concurrent inhibition of amylase, resulting in an increase in undigested carbohydrates in the colon [7]. Additionally, some of them may contribute to the incidence of acute hepatitis, hepatic injury, and renal tumors [8]. To resolve this issue, research for natural phytochemicals that have been known to attenuate α-glucosidase activity and have lower side effects than synthetic drugs has been conducted [8,9]. Some classes of plant phytochemicals have been reported as having the capability to attenuate α-glucosidase activity, including phenolic acids [8], flavonoids [10], terpenoids or essential oils [11], etc.

*Persicaria odorata* (Lour.) is an important herbal remedy that is utilized in traditional medicine to alleviate edema and inflammation, diarrhea, excessive hemorrhaging, ulcers, lesions, and wounds [12]. It has been traditionally consumed as a fresh vegetable in Southeast Asian cuisine. Meanwhile, it is employed as a traditional Thai remedy to alleviate dyspepsia and as a flavoring agent in Thailand [13]. The pharmacological benefits of *P. odorata* were sourced from its rich phytochemical contents [14], such as phenolics, which are categorized as phenolic acids and flavonoids [15,16,17], and essential oils or terpenoids [18,19,20]. *P. odorata* is known to contain high total phenolic and flavonoid contents in leaf and stem extracts, especially methanolic extract, which was linked to its antioxidant capability to scavenge free radicals at the molecular level [13,21]. Some previous studies have demonstrated that the ethanolic extract of leaves and stems [18] and the water extract of leaves [19] of *P. odorata* attenuated α-glucosidases activity. Unfortunately, even though phytochemical compounds were elucidated, the specific bioactive compounds responsible for the α-glucosidase inhibitory activity have not yet been elucidated. Therefore, this study aims to determine the methanolic extract of *P. odorata* on α-glucosidase inhibitory activity, and to investigate the bioactive compounds in *P. odorata* extracts as α-glucosidase inhibitors via integrated in vitro and in silico studies.

## 2. Results and Discussions

### 2.1. α-Glucosidase Inhibitory Activity

The α-glucosidase enzyme is a therapeutic target for T2DM since α-glucosidase inhibitors play a vital role in preventing the breakdown of carbohydrates to simple glucose (α-D-glucose), resulting in control of postprandial hyperglycemia in T2DM. Currently, natural products have attracted much attention as a kind of potentially safer α-glucosidase inhibitors [22]. Therefore, this study was conducted to determine the potential activity of the methanolic extract of *P. odorata* by in vitro studies and to explore its bioactive compounds as α-glucosidase inhibitors by in silico studies.

In this study, yeast α-glucosidase with 4-nitrophenyl-α-D-glucopyranoside as the substrate was used to initially evaluate the extract’s inhibitory activity. In addition to being widely used and cost-effective, yeast α-glucosidase catalyzes the same reaction as human α-glucosidase [23].

**Figure 1:**
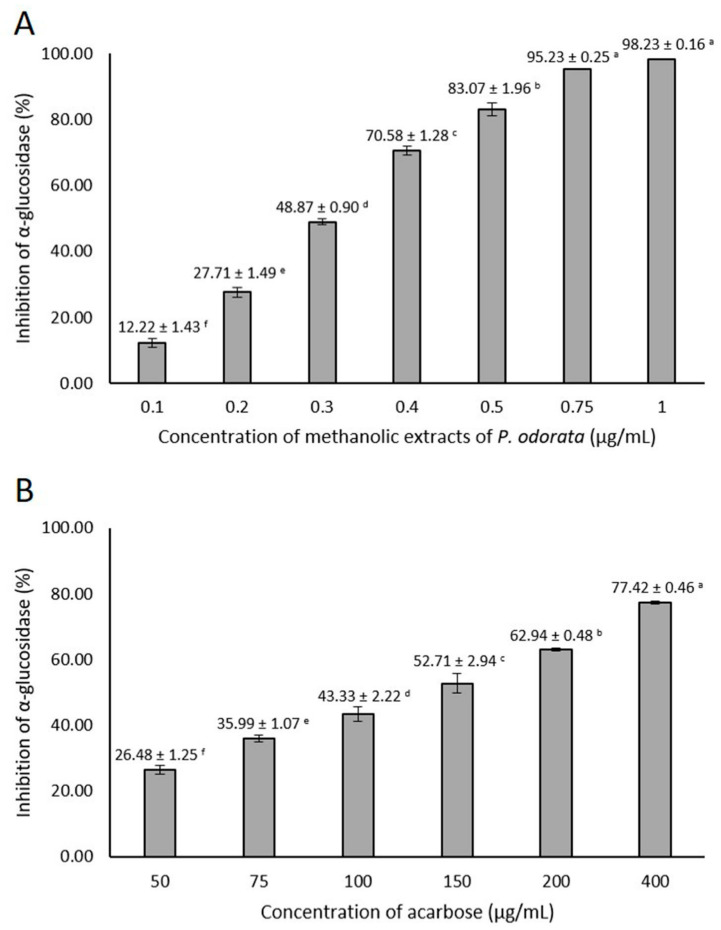
The inhibition percentage of (**A**) methanolic extract of *P. odorata* and (**B**) acarbose on the α-glucosidase activity. Data are shown as mean ± SD (n = 3). Superscript ^a–f^ means the different superscript letters in each bar are significantly different (*p* < 0.05).

Inhibition of yeast α-glucosidase reflects the in vitro antidiabetic potential determined by spectrophotometry. The enzyme activity assay was conducted while taking care to avoid factors that could interfere with the enzyme inhibitory activity assay, following our protocol, including temperature, time, chemicals, and technical personnel. In the current study, the inhibition percentage values of the methanolic extract of *P. odorata* compared with the standard drug acarbose for yeast α-glucosidase activity are presented in Figure 1. Even though the extract contains many phytoconstituents, it could be used to assess its potential activity relative to acarbose. The in vitro results exhibited that the methanolic extract of *P. odorata* had a strong capability for the yeast α-glucosidase inhibition (IC_50_ = 0.31 ± 0.00 µg/mL) in a dose-dependent manner, which was 450-fold more potent than acarbose (IC_50_ = 139.47 ± 5.04 µg/mL), under experimental conditions. Further statistical analysis also indicated that the methanolic extract of *P. odorata* and acarbose were significantly different in inhibiting the yeast α-glucosidase activity (*p* < 0.05). The excellent inhibitory activity may be attributed to the phytochemicals of the crude extract, as *P. odorata* is known to contain high levels of phytoconstituents, so the synergistic or additive inhibition might be expected against the yeast α-glucosidase [24,25]. Previous researchers, Kee et al. [26] reported that the water extract of *P. odorata* has potentially inhibited both yeast and rat intestinal α-glucosidase, which was linked to an early study reporting that *P. odorata* inhibited both digestive enzymes, α-amylase and α-glucosidase [21]. However, due to several differences in size and amino acid sequences among yeast, rat, and human intestinal α-glucosidases, additional research linking rat and human intestinal enzymes to cell-based experiments is necessary to confirm the antidiabetic activity of the extract [27]. Moreover, further studies focused on the fractionation or isolation of pure compounds from the *P. odorata* extract are recommended.

### 2.2. Enzyme Kinetics Study Analysis

**Figure 2:**
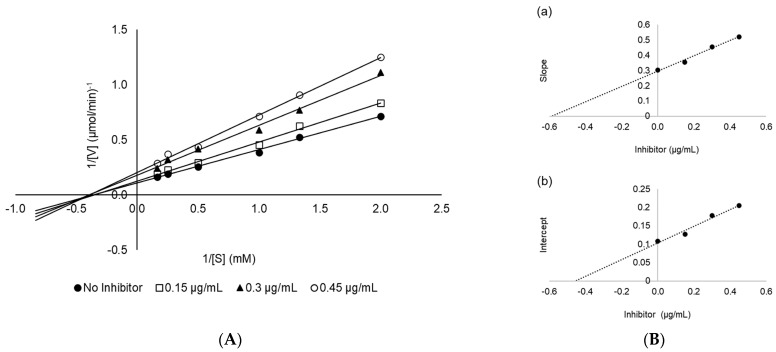
(**A**) Lineweaver−Burk plots of the kinetics analysis of α-glucosidase in the presence of the methanolic extract of *P. odorata*. (**B**) The slope (**a**) and intercept (**b**) of the Lineweaver–Burk plots against inhibitor concentrations. The substrate concentration was measured in the absence or presence of extracts at different concentrations. [V] and [S] are the reaction velocity and substrate concentration, respectively. (Dark circles: no inhibitor, black-lined transparent squares: 0.5IC_50_ concentration, dark triangles: IC_50_ concentration, black-lined transparent circles: 1.5IC_50_ concentration).

Enzymes exhibit varying inhibitor affinity, as evidenced by their inhibition constants. The dissociation constant (*K*_i_) was obtained from the secondary plot of the slope of the linear relationships of 1/[V] vs. 1/[S] in relation to the concentration of inhibitors. The *K*_i_ value is determined by the intercept on the inhibitor axis of this plot. The *K*_ii_ value of the inhibitor constant was determined by plotting 1/V_max_ against the inhibitor concentration in a linear secondary plot. The *K*_ii_ values are determined by the intercept of the inhibition axis [28]. Since the methanolic extract of *P. odorata* had strongly inhibited α-glucosidase activity, the enzyme kinetics study was performed to identify the type of inhibition of the extract. The hydrolysis reaction catalyzed by α-glucosidase was observed at a variety of substrate concentrations [S] (0.5, 0.75, 1, 2, 4, and 6 mM) to determine the initial velocity [V_0_] in the absence and presence of the extracts (0.15, 0.3, 0.45 µg/mL). In this current study, Lineweaver–Burk plots indicated that the methanolic extract of *P. odorata* inhibited the α-glucosidase with a noncompetitive inhibition (Figure 2). Noncompetitive inhibition means the inhibitor binds both the free enzyme (E_0_) and the enzyme–substrate (ES) complex at a different site on the enzyme [29]. Most noncompetitive inhibitors are chemically unrelated to the substrate, so increasing substrate concentration would not overcome the inhibition. In addition, noncompetitive inhibitors can decrease the concentration of active enzymes in the solution, thereby decreasing the V_max_ of the reaction; however, they do not influence the *K*_m_ value [30]. A noncompetitive inhibitor interacts with the enzyme at a location distinct from the actual active site. Consequently, the inhibitor’s binding does not physically obstruct the substrate binding site but can prevent subsequent reactions. An uncompetitive inhibitor binds exclusively to the ES complex, not to the free enzyme, and at a site distinct from the substrate’s active site [30].

According to *K*_m_ and V_max_ values obtained from Lineweaver–Burk plots, it was found that *K*_m_ values remain constant, and V_max_ values are decreased. In this study, α-glucosidase had a *K*_m_ value of 2.797 mM for *p*NPG and a V_max_ value of 9.259 µmoles. In the presence of 0.15, 0.30, and 0.45 µg/mL of the extracts, apparent V_max_ values were found to be 7.874, 5.612, and 4.885 µmoles, and *K*_m_ values remained constant at 2.791, 2.547, and 2.546 mM, respectively (Table 1). These findings indicated that the velocity [V] of the reaction catalyzed by α-glucosidase was influenced by the binding of the bioactive compounds of the extracts in a manner that was proportional to the concentration of the extracts in the reaction mixture, while the *K*_m_ remained unaffected [30]. This result is linked to an early study stating that the ethanolic extract of *P. odorata* leaves and stems exhibited noncompetitive inhibition of α-glucosidase [21]. In contrast, acarbose is well known for inhibiting α-glucosidase competitively [3,29].

### 2.3. Bioactive Compounds Contained in P. odorata Extract

*P. odorata* contains high levels of phenolics and essential oils. In this study, there were seven phenolics from the methanolic extract of *P. odorata* positively detected by our group using HPLC-DAD-MS, including chlorogenic acid, sinapic acid, caffeic acid, gallic acid, protocatechuic acid, rutin, and quercetin. These compounds were selected because they are commonly found in the plant. On the other hand, as *P. odorata* contained high levels of phytochemicals, other bioactive compounds within any *P. odorata* extracts from the previous reports were selected to investigate the α-glucosidase inhibitors. A total of forty-four bioactive compounds were selected, which were divided into two main groups, namely phenolics and essential oils. Phenolics consisted of 23 compounds, including seven phenolic acids and 16 flavonoids identified by HPLC-DAD-MS, and 21 essential oils identified by GC-MS (Table 2). There were more phytochemicals found within those *P. odorata* extracts than in this study, especially in the essential oil group. Only essential oils with an amount higher than 1% or specifically identified were selected [18,19,20]. Table 2 presents the complete list of these compounds, while their corresponding chemical structures are depicted in Figure S1 (phenolics) and Figure S2 (essential oils). In addition, the chromatograms of phenolics in the methanolic extract of *P. odorata* detected by our group are presented in Figures S3–S5.

### 2.4. Molecular Docking

**Figure 3:**
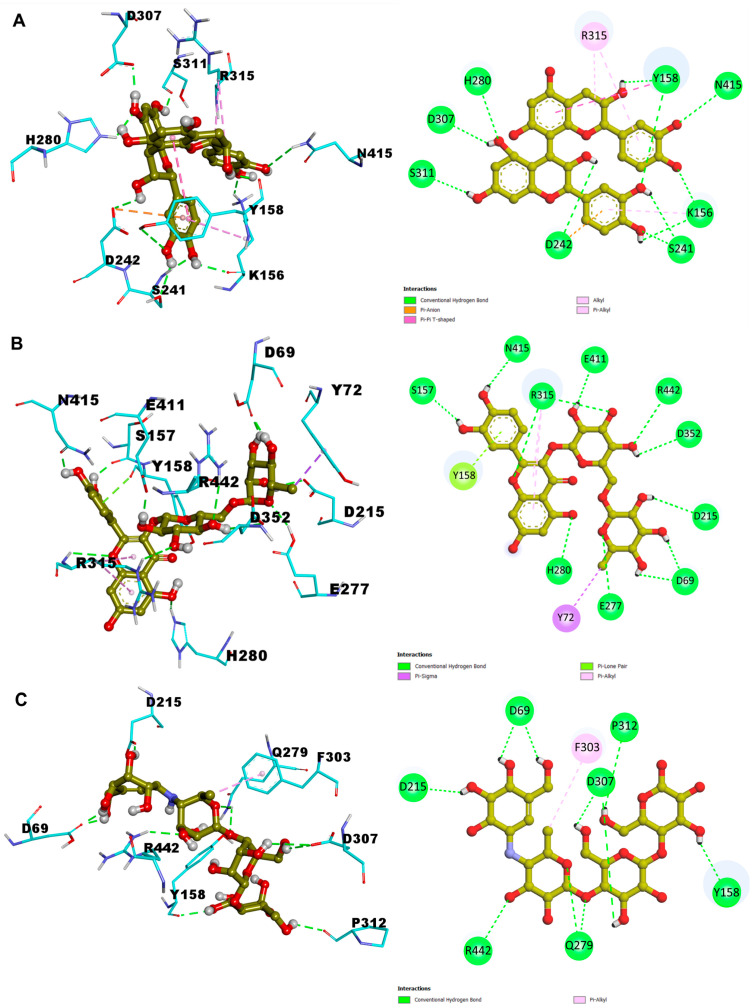
The visualizations of the molecular docking interactions on the α-glucosidase’s active site. 3D visualizations (**left**), 2D visualizations (**right**), (**A**) P9, (**B**) P19, and (**C**) acarbose.

In vitro studies have demonstrated that the methanolic extract of *P. odorata* exhibits promising inhibitory activity against α-glucosidase, consistent with previous findings [21,26]. To further investigate this inhibitory mechanism, a molecular docking study was conducted using AutoDock4 on various bioactive compounds present in the extracts, including 23 phenolics and 21 essential oils. AutoDock4 is an excellent non-commercial docking program that is widely used. Further, it employs a stochastic Lamarckian genetic algorithm to compute ligand conformations and simultaneously minimize its scoring function, which approximates the thermodynamic stability of the ligand bound to the target protein [34]. The binding energy (Δ*G*) scores, inhibition constant (*K*_i_) scores, hydrogen bond (H-bond) formations, and hydrophobic interactions between these phenolics, essential oils, and the standard drug acarbose with the amino acids of α-glucosidase’s active site are summarized. Acarbose exhibited a Δ*G* score of −7.30 kcal/mol. Notably, ten phenolics displayed Δ*G* scores lower than acarbose, including **P7**–**P9**, **P11**, **P12**, **P14**–**P16**, **P19**, and **P20**. Among these, **P9** and **P19** emerged as the most promising candidates, with the lowest Δ*G* scores of −9.90 kcal/mol and −9.42 kcal/mol, respectively (Table 3). Interestingly, only compound **O7** from the essential oil group demonstrated the Δ*G* of −7.41 kcal/mol, which was lower than that of acarbose. Figure 3 shows the representative molecular docking interactions with amino acids of α-glucosidase in 3D and 2D visualizations of **P9** and **P19** compared to acarbose.

In the analysis of molecular binding interactions, **P9** formed eight H-bonds, including K156, Y158, S241, D242, H280, D307, S311, and N415. In addition, hydrophobic interactions were formed by K156, Y158, D242, and R315 residues. Meanwhile, **P19** formed 10 H-bonds, including D69, S157, D215, E277, H280, R315, D352, E411, N415, and R442. Moreover, Y72, Y158, and R315 residues also formed hydrophobic interactions. According to these results, although **P9** has fewer H-bonds than **P19**, it was found that **P9** formed double bonds via H-bonds with three amino acid residues, including K156, Y158, and S241. Meanwhile, **P19** formed double bonds via H-bonds with two residues, including D69 and R315. Additionally, **P9** interacted via hydrophobic interactions with four residues, while **P19** interacted with three. Consequently, **P9** has a higher binding affinity than **P19** (Table 3 and Figure 3).

Compared to a standard drug, acarbose formed seven H-bonds with amino acid residues, including D69, Y158, D215, Q279, D307, P312, and R442, and a hydrophobic interaction with the F303 residue. This molecular docking result demonstrated that **P9** shared similar interacting residues with acarbose, such as Y158 and D307, while **P19** shared similar interacting residues with acarbose, which are D69, D215, and R442. These interactions indicated that **P19** preferred interacting with a similar site pocket to acarbose, while **P9** preferred interacting with different sites from acarbose on α-glucosidase. For more in-depth analysis, molecular dynamics simulations were conducted, and the stability of **P9**, **P19**, and acarbose with α-glucosidase enzyme affinity will be discussed below.

On the other hand, for the essential oil group, **O7** formed three H-bonds with a single bond via K156, L313, and R315 residues. In addition, residues L156 and Y158 were found to form hydrophobic interactions. Most essential oils formed hydrophobic interactions and lacked H-bonds with amino acids of the active site of α-glucosidase, causing them to have low binding affinity (Table 4). The molecular interaction among other phenolics and essential oils with the amino acids of α-glucosidase’s active site in 2D visualization can be found in the Supplementary Materials, Figures S6 and S7. It is known that H-bonds have a stronger binding affinity than hydrophobic interactions. Nevertheless, it has been considered that hydrophobic interactions contributed to protein stability and enhanced the binding affinity and biological activity of the ligand–receptor complex [35,36]. Further in vitro study of this **O7** compound against α-glucosidase is needed.

### 2.5. Molecular Dynamics Simulations Analysis

**Figure 4:**
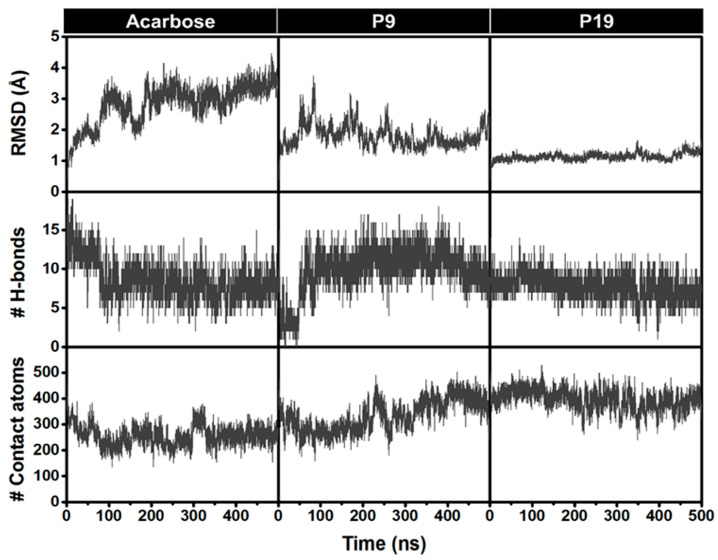
RMSD of the backbone atoms, the number of H-bonds, and the number of atom contacts for acarbose, P9, and P19 in complex with α-glucosidase along the 500 ns MD simulations.

The dynamic behavior of potent compounds **P9** and **P19** bound to α-glucosidase was investigated using 500 ns MD simulations, compared to that of the positive control acarbose. To assess the stability of protein–ligand complexes, RMSD (root mean square deviation) of the backbone atoms within 10 Å of the active site, the # H-bonds, and the # atom contacts were calculated throughout the simulation time (Figure 4). Both acarbose and the **P9** system exhibited an initial increase in RMSD values, followed by small fluctuations at ~2.5–3.5 Å and ~1.5–2.5 Å, respectively, for the remainder of the simulation. In contrast, the **P19** system remained stable during the 500 ns simulation (RMSD ~1.0–1.5 Å). Over the last 100 ns, the average # H-bonds formed by acarbose, **P9**, and **P19** were 8 ± 2, 10 ± 2, and 7 ± 1, respectively. The # atom contacts for these inhibitors ranged from ~257–401 atoms, with **P9** exhibiting the highest # atom contacts, aligning with its lowest binding energy observed in the molecular docking study. This suggests that **P9** could interact more favorably with α-glucosidase than the other inhibitors.

**Figure 5:**
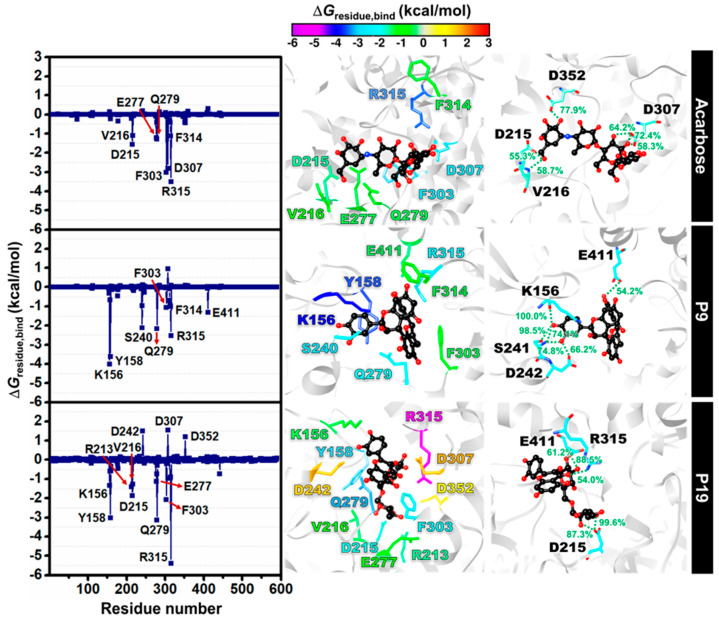
The Δ*G*_residue,bind_ values of inhibitor-α-glucosidase complexes are depicted. Note: Residues contributing to inhibitor binding are colored according to their Δ*G*_residue,bind_ values, with the highest free energy residues colored red and the lowest free energy residues colored purple. The representative structure is used to illustrate the binding pattern of the inhibitor-α-glucosidase complexes. Green dashed lines represent H-bond formation.

The key residues involved in inhibitor binding to α-glucosidase were identified using the MM/GBSA method (Figure 5, left). The binding orientations of each inhibitor with the active site of α-glucosidase, with hot-spot residues colored according to their Δ*G*_residue,bind_ values, are shown in Figure 5 (middle). Only residues exhibiting significant energy stabilization or destabilization (<−1.0 and >1.0 kcal/mol) are labeled and discussed. It was found that **P19** interacted with 12 key residues, including K156, Y158, R213, D215, V216, D242, E277, Q279, F303, D307, R315, and D352. Acarbose interacted with eight key residues: D215, V216, E277, Q279, F303, D307, F314, and R315. Similarly, **P9** interacted with eight key residues: K156, Y158, S240, Q279, F303, F314, R315, and E411. Both acarbose and **P19** bind within a hydrophobic pocket formed by the D215 and E277 residues, similar to the previously reported binding mode of ursolic acid [37]. Notably, the residues Q279, F303, and R315 played crucial roles in the binding of all the compounds. Additionally, D215, a catalytic residue involved in maltose hydrolysis [38], contributed to the stabilization of both acarbose and **P19** within the binding pocket through H-bonds.

To further elaborate on the insight, the percentage of H-bond occupations formed between the inhibitors and α-glucosidase residues was monitored and shown in Figure 5 (right). While H-bonds exceeding 50% occupancy were initially selected for analysis, only those exceeding 70% occupancy are discussed here to focus on strong stabilization. For the acarbose system, two strong H-bonds were identified with the residues D307 (72.4%) and D352 (77.9%). The **P9** system also formed four strong H-bonds with K156 (100%) and S241 (98.5, 74.8, and 74.1%). Additionally, the **P19** system exhibited three strong H-bonds with D215 (99.6 and 87.3%) and E411 (88.5%). Interestingly, the H-bonding pattern between D215 and acarbose and **P19** closely resembled that observed during maltose binding to α-glucosidase [38]. These observed H-bonding interactions suggest that acarbose and **P19** may inhibit α-glucosidase by binding in a manner similar to that of maltose, thereby effectively reducing the enzyme’s activity. Moreover, to confirm this activity, the promising in silico approach results, **P9** and **P19**, were then subjected to further in vitro and in vivo investigations. It should be noted that a single 500 ns MD trajectory was performed for each protein–ligand complex in the present study. Although all systems reached stable conformations and multiple analyses consistently supported the proposed interaction mechanism, independent replicate simulations would further strengthen the statistical robustness of dynamic properties and binding free-energy estimates. To characterize protein–ligand interactions and identify key residues contributing to substrate recognition, MM/GBSA analysis was performed as previously reported [DOI: 10.1038/s41598-025-24941-5, DOI: 10.1080/07391102.2023.2201360, doi.org/10.1007/s10562-026-05443-z]. Because the aim was to assess relative interactions rather than calculate absolute binding affinities, entropy corrections were not included.

### 2.6. Drug-Likeness and ADMET Property Analysis

Drug-likeness and ADMET properties were predicted using computational approaches, such as SwissADME and pkCSM online software, respectively, to determine the probability of the promising bioactive compounds in *P. odorata* extracts for the development of drugs, especially α-glucosidase inhibitors. Lipinski’s Rule of Five (Ro5) predicted the physicochemical properties of the compounds [39], while ADMET assessed the pharmacokinetics of the compounds.

The predicted results demonstrated that **P9** and **P19** did not pass Lipinski’s rule because they have three Lipinski’s Ro5 violations, including high molecular weight and numerous hydrogen-bond donors and acceptors, similar to acarbose (Table 5). This may lead to poor absorption from the small intestinal tract into the systemic circulation. However, this consequence would not affect compounds targeting α-glucosidase inhibition, which are abundant on the brush border of the small intestine [40]. Additionally, 12 phenolics passed Lipinski’s Ro5, including **P2**–**P6**, **P8**, **P17**, **P18**, **P20**–**P22**, and **T1**, while the other 11 phenolics did not pass Lipinski’s Ro5, including **P1**, **P7**, **P9**–**P16**, and **P19**. Most phenolics did not pass Lipinski’s Ro5 because their H-bond acceptors or donors and topological polar surface area (TPSA) were higher than standard values. Meanwhile, 13 essential oils were predicted to pass Lipinski’s Ro5, including **O1**, **O2**, **O4**–**O8**, **O15**–**O18**, **O20**, and **O22**. The other eight essential oils that did not pass Lipinski’s rule were **O3**, **O9**–**O14**, and **O19** due to their Mlog*p* values (>4.15). More details on drug-likeness predictions for phenolics and essential oils can be found in Table S1.

According to ADMET property predictions (Table 5), **P9** and **P19** were predicted to have lower water solubility and Caco2 permeability than acarbose. In human intestinal absorption (HIA), **P9** could be absorbed in the human intestine (66.7%), which may reduce the concentration available to inhibit α-glucosidase, while **P19** and acarbose were poorly absorbed, suggesting that **P19** and acarbose may have a long therapeutic duration. Generally, these properties might suggest **P9** and **P19** to be poor candidates for systemic agents [39]. However, for intestinal α-glucosidase inhibitors, these properties may actually be advantageous and consistent with the mechanism of action [40]. HIA is an essential parameter for developing an α-glucosidase inhibitor because it provides information on how much a drug is absorbed in the human small intestine, where α-glucosidases are abundant. In addition, **P9**, **P19**, and acarbose were predicted as P-glycoprotein (P-gp) substrates. Consequently, P-gp could detect and re-efflux them from the cells back into the lumen [41]. Interestingly, **P9** was also predicted to be a P-gp inhibitor I and II, whereas **P19** and acarbose were not P-gp inhibitors I and II [42], meaning **P9** could mitigate P-gp efflux and improve the oral absorption and bioavailability of several P-gp substrates [43]. Other ADMET properties of phenolics and essential oils can be found in Tables S2–S6.

The distribution properties predicted that **P9** and acarbose had a low volume of distribution (VD). In contrast, **P19** had a high VD, meaning that **P9** would distribute to the plasma, while **P19** would distribute instead to the tissues rather than the plasma. The blood–brain barrier (BBB) predicted that **P9**, **P19**, and acarbose were poorly distributed to the brain. The results predicted that most phenolics did not pass the BBB. Conversely, most essential oils could pass the BBB.

**P9** and **P19**, as well as acarbose, were not substrates and inhibitors of cytochrome P450 isoforms. **P9** and **P19** were predicted to have lower total clearance than acarbose. **P9** was a renal organic cation transporter-2 (OCT2), while **P19** and acarbose were not renal OCT2. OCT2 is crucial for the renal clearance of drugs and the disposition of drugs in the kidney. Regarding toxicity, **P9** and **P19** were not carcinogens, were not hERG I inhibitors but hERG II inhibitors, and were safe for the liver, with properties similar to acarbose [42]. This indicates that, even though **P9** was predicted to have much higher HIA than **P19** and acarbose, it remains safe for systemic exposure. Therefore, these predicted results suggest that **P9** and **P19** may be potentially developed as α-glucosidase inhibitors.

## 3. Materials and Methods

### 3.1. Chemicals and Reagents

4-nitrophenyl-α-D-glucopyranoside or *p*NPG (Tokyo Chemical Industry Co., Ltd., Tokyo, Japan), the α-glucosidase enzyme (Megazyme Ltd., Wicklow, Ireland), acarbose (Fujifilm Wako Pure Chemical Corporation, Osaka, Japan), sodium bicarbonate (Na_2_CO_3_), methanol, and dimethyl sulfoxide (DMSO) were purchased from Sigma-Aldrich^®^ Co. Ltd. (St. Louis, MO, USA). Phosphate buffer (100 mM, pH 6.8) was prepared using sodium dihydrogen phosphate (NaH_2_PO_4_·H_2_O) and disodium hydrogen phosphate anhydrous (Na_2_HPO_4_). Distilled water (≤18.2 mΩ) was produced using an ELGA Purelab Option-Q 15 BP (Chemoscience, Bangkok, Thailand).

### 3.2. Plant Collection and Extraction

*P. odorata* plants were purchased from the market of Maha Sarakham, Maha Sarakham Province, Thailand, in January 2023. The plant was identified by Assistant Professor Dr. Wanida Caichompoo of the Faculty of Pharmacy at Mahasarakham University. Voucher specimen was deposited at the Herbarium, Faculty of Pharmacy, Mahasarakham University with No. MSU.PH-POL-PO01. The leaves and stems were collected and washed with water, dried for one day in a heated-air oven at 45 °C, and powdered using an iron mortar and pestle before extraction. Approximately 100 g of the powdered plant was used and extracted with methanol at a 1:2 (*w*/*v*) ratio by sonication for 30 min [44]. Then, the resulting extracts were filtered with Whatman No. 1 filter paper, and the residue was extracted twice. The filtrate was desiccated at 40 °C using a rotary evaporator (Rotavapor^®^ R-100, Buchi, Flawil, Switzerland). This extraction process yielded a value of 10.42%. Lastly, the filtrates were chilled and stored in securely sealed glass at 4 °C until analysis. All experiments, including the phytochemical screening and biological assays, were conducted using extracts from the same batch.

### 3.3. α-Glucosidase Inhibitory Activity Assay

The α-glucosidase inhibitory assay was conducted spectrophotometrically, with a few adjustments to the previously described procedure [45]. Briefly, 50 µg/mL of methanolic extract of *P. odorata* was dissolved with DMSO 5% (the final concentration was less than 1%), which did not affect enzyme activity, then mixed with 15 μL (0.5 U/mL) of the α-glucosidase solution and 20 μL of phosphate buffer (100 mM; pH 6.8). A 50 μL volume of the extract at varying concentrations (0.1, 0.2, 0.3, 0.4, 0.5, 0.75, and 1 μg/mL) was inserted into a 96-well microplate. Another 10 min was devoted to incubating the reaction mixture at 37 °C. Subsequently, 15 μL of *p*NPG (5 mM) was incorporated into the reaction mixture to initiate the enzymatic reaction and incubated at 37 °C for 15 min. Immediately, the reaction mixture was supplemented with 150 μL of Na_2_CO_3_ (1 M) solution to retard the enzymatic activity. In place of the sample, DMSO was employed to produce the control. Similarly, by changing the enzyme and *p*NPG with buffer, blank samples were generated for each sample. In order to quantify the absorbance of *p*-nitrophenol that was emitted during the reaction, the microplate reader was employed to measure at 405 nm. Acarbose, as an α-glucosidase inhibitor agent, was implemented as a positive control. The inhibition percentage of the α-glucosidase activity was determined by employing the following formula:

$$Inhibition\left(\%\right)=\frac{Absorbance\;of\;Control-Absorbance\;of\;Sample}{Absorbance\;of\;Control}\times 100$$

### 3.4. Enzyme Kinetics Study

The detailed procedure for the enzyme kinetics study of the α-glucosidase by the methanolic extract of *P. odorata* was also similar to the α-glucosidase inhibition assay described above. In order to evaluate the reversibility of enzyme inhibition, the reaction rate was calculated by increasing the substrate concentration, *p*NPG (0.5, 0.75, 1, 2, 4, and 6 mM), in the absence or presence of inhibitor, methanolic extract of *P. odorata*, at three various concentrations (0.15, 0.3, and 0.45 µg/mL). The enzyme inhibitory reaction was recorded by measuring the absorbance of the microplate reader at 405 nm for 60 min at 37 °C. To ascertain the type of enzyme inhibition induced by inhibitors, the Lineweaver–Burk plot was employed to determine the V_max_ and *K*_m_, which were calculated from the results according to Michaelis–Menten kinetics [46]. From the secondary plots of the primary rate data, the enzyme inhibitor [EI] and enzyme substrate inhibitor [ESI] constants were determined through linear regression analysis. *K*_i_ values were determined by plotting the slopes of the Lineweaver–Burk plots against inhibitor concentrations. In contrast, *K*_ii_ values were determined by plotting intercepts against inhibitor concentrations derived from the x-intercepts [28].

### 3.5. HPLC-DAD Analysis

High-performance liquid chromatography (Shimadzu Co., Tokyo, Japan) with a UV diode-array detector (HPLC-UV-DAD) was used to determine the phenolic profile of the methanolic extract of *P. odorata*. Analysis of the phenolic profile was performed as described in our previous study [47]. The extract was filtered using a 0.45 µm filter. The separations were realized using a Unisol C18 column as a stationary phase (4.6 mm inside diameter × 250 mm length, 5 µm particle size). Gradient elution was performed at a constant flow rate of 0.8 mL min^−1^, with Acetic acid 1% (A) and acetonitrile 100% (B) as the mobile phase. The spectral data were acquired at 280, 320, and 380 nm for UV detection. The retention times of the extract were identified by comparing retention times with standard phenolic acids, including gallic acid, protocatechuic acid, syringic acid, p-hydroxybenzoic acid, and caffeic acid at 280 nm, chlorogenic acid, p-coumaric acid, ferulic acid, and sinapic acid at 320 nm, and rutin and quercetin at 370 nm. The standard phenolics and extract were analyzed in triplicate. The spectra of each compound and the chromatogram are shown in Figures S3–S5.

### 3.6. Statistical Analysis

Each experiment was conducted in triplicate, and the results were reported as the mean ± standard deviation (SD). The statistical analysis was calculated utilizing IBM28 SPSS (version 28, Armonk, New York, NY, USA). The one-way analysis of variance (ANOVA) was implemented to assess the variations among the extracts, which was subsequently followed by Tukey’s test. A significant difference was defined as a *p*-value (*p* < 0.05).

### 3.7. Data Collection of Bioactive Compounds Contained in P. odorata Extracts

Phytochemical compounds of *P. odorata* extracts were obtained carefully from previous researchers’ reports who had screened, identified, and isolated bioactive compounds from *P. odorata* extracts. There were 44 compounds collected and divided into two main groups, including 23 phenolics and 21 essential oils, where phenolics consist of seven phenolic acids and 16 flavonoids. Only in silico approaches were applied for these 44 compounds to predict their binding affinity with α-glucosidase by molecular docking, then the two lowest binding affinities were chosen for analyzing their binding stability by molecular dynamics simulations, and their pharmacokinetic properties were predicted.

### 3.8. Molecular Docking Study

The methanolic extract of *P. odorata* exhibited potent inhibitory activity against α-glucosidase. To elucidate the molecular basis of this inhibition, molecular docking studies were performed using a comprehensive set of previously characterized bioactive compounds derived from this plant. Due to the unavailability of the three-dimensional (3D) structure of *Saccharomyces cerevisiae* α-glucosidase, the 3D crystal structure of the *S. cerevisiae* isomaltase (PDB ID: 3A4A), which has a resolution of 1.60 Å and shares high sequence identity with 72% identity and 85% similarity with *S. cerevisiae* α-glucosidase, was retrieved from the RCSB Protein Data Bank (PDB) [48,49]. The higher resolution of the receptor provides an effective receptor file [50], and the high degree of sequence homology suggests that this structure is a suitable and reliable surrogate for *S. cerevisiae* α-glucosidase in molecular docking studies, as demonstrated in several previous studies [49,51,52,53].

Prior to docking calculations, the protein’s 3D structure was properly prepared. Firstly, the protein and ligand were protonated at pH 7.4 to simulate physiological conditions using PDB2PQR [54] and Marvin, respectively. The identified compounds were retrieved from the PubChem database (https://pubchem.ncbi.nlm.nih.gov (accessed on 14 February 2026)). AutoDock Tools version 1.5.7 (ADT v1.5.7) was used to prepare both the protein and compound structures [34]. Second, all water molecules, ligands, and heteroatoms were eliminated from the protein. Polar hydrogens and Kollman charges were subsequently assigned to the protein [55], and missing and terminal residues in polypeptide chains were repaired using ADT v1.5.7. It was ensured that no residues contained non-integral charges, a requirement for the receptor file in AutoDock4.2. The preparation of both ligands and receptors was saved as PDBQT files for the docking simulations software [50]. The center of the predicted active site was defined as the center of a 60 × 60 × 60 Å grid box with a 0.375 Å grid spacing. The grid box was centered at coordinates X = 21.519, Y = −7.702, and Z = 23.554. Compound structures were subsequently optimized using ADT and saved in PDBQT format. Molecular docking simulations were then performed using AutoDock 4.2.6 with the Lamarckian Genetic Algorithm (LGA) method, with 100 independent runs, a population size of 150, an energy evaluation of 25 × 10^5^, while all other parameters were kept at their default values [34]. The docking protocol was validated by re-docking the native ligand into the α-glucosidase active site. The re-docking RMSD value of 0.31 Å, which was less than 1 Å, confirmed the reliability of the protocol [56,57]. The docked conformation with the lowest binding energy was selected for subsequent interaction analysis, including hydrogen-bond and hydrophobic interactions, using BIOVIA Discovery Studio Visualizer 2021.

### 3.9. Molecular Dynamics Simulations

To explore the structural and dynamic behavior of a compound against α-glucosidase in an aqueous solution, all-atom MD simulations were conducted with periodic boundary conditions (PBC) using the AMBER22 software package. Initially, the selected compounds underwent optimization via the Gaussian09 program at the HF/6-31G(d) level, followed by the generation of their restrained electrostatic potential (RESP) charges using the antechamber module of AMBER22, according to our standard protocol [58,59]. The protein and ligand were assigned to the AMBER ff19SB force field [60] and the generalized AMBER force field 2 (GAFF2) [61], respectively. Subsequently, missing hydrogen atoms were added to the protein–ligand complex using the leap module. Next, the system was solvated with a TIP3P water model [62] and subjected to further minimization, first with restraints on the solvent and then with full system minimization. Non-bonded interactions were set at a 12 Å cutoff, and long-range electrostatic interactions were treated with the Particle Mesh Ewald method [63]. The target pressure (1 atm) was maintained using the Berendsen barostat [64], while covalent bonds involving hydrogen atoms were constrained using the SHAKE algorithm [65]. The system was heated up to 310 K for 100 ps, followed by a 100 ps equilibration period using the Langevin thermostat [66] with a collision frequency of 2.0 ps^−1^. Finally, 500 ns MD simulations of the complex systems were conducted at 310 K and 1 atm. The structural analysis focused on various parameters, including root mean square deviation (RMSD), intermolecular hydrogen bonding (# H-bonds), and the number of contact atoms (# Contact atoms), which were calculated using the CPPTRAJ module [67]. Additionally, the binding free energy decomposition per residue (Δ*G*_residue,bind_) was determined using the Molecular Mechanics Generalized Born Surface Area (MMGBSA) [68] method based on 100 snapshots from the last 100 ns of the simulations.

### 3.10. ADMET Property Prediction

Both online servers of SwissADME (http://www.swissadme.ch/index.php (accessed on 14 February 2026)) was used to analyze the drug-likeness Lipinski’s rule of 5 (Ro5) and pkCSM (http://biosig.unimelb.edu.au/pkcsm/ (accessed on 14 February 2026)) was used to predict pharmacokinetic properties, which are absorption, distribution, metabolism, excretion, and toxicity (ADMET) of the bioactive compounds within *P. odorata* extracts using their Simplified Molecular Input Line Entry System (SMILES) [42,69].

## 4. Conclusions

The methanolic extract of *P. odorata* exhibited strong inhibitory activity against yeast α-glucosidase enzyme compared to acarbose, an α-glucosidase inhibitory agent. The type of inhibition suggests a noncompetitive inhibitor, indicating that its bioactive compounds predominantly bind to the enzyme [E_0_] and to the enzyme–substrate [ES] complex at sites distant from the substrate binding site, confirming that *P. odorata* could be used as an alternative edible plant to control postprandial hyperglycemia in T2DM. On the other hand, procyanidin B (**P9**) and rutin (**P19**), which belong to the flavonoids, were predicted by computational approaches to be bioactive compounds that have more favorable binding affinity to yeast α-glucosidase residues. Moreover, both **P9** and **P19** were predicted to qualify as α-glucosidase inhibitors with low toxicity. These findings could serve as basic scientific data, and further in vitro and in vivo studies are necessary to support these findings.

## Figures and Tables

**Table 1:** Enzyme kinetic parameters of the methanolic extract of *P. odorata* are presented as *K*_m_, V_max_, *K*_i_, *K*_ii_, and *K*_ii_/*K*_i_ values.

Extract (μg/mL)	*K*_m_ (mM)	V_max_ (μmol/min)	*K*_i_ (µg/mL)	*K*_ii_ (µg/mL)	*K*_ii_/*K*_i_ (µg/mL)
0	2.797	9.259	0.29	0.10	0.34
0.15	2.791	7.874			
0.30	2.547	5.612			
0.45	2.546	4.885			

**Table 2:** Bioactive compounds within *P. odorata* (Lour.) extracts obtained from previous reports.

Phenolics					
Groups	Compounds	Formula	Codes	PubChem NID ^1^	References
Phenolic acids	Chlorogenic acid ^2^	C_16_H_18_O_9_	P1	1794427	[16,17]
	Sinapic acid ^2^	C_11_H_12_O_5_	P2	637775	
	Caffeic acid ^2^	C_9_H_8_O_4_	P3	689043	
	Gallic acid ^2^	C_7_H_6_O_5_	P4	370	
	Protocatechuic acid ^2^	C_7_H_6_O_4_	P5	72	
	Methyl gallate	C_8_H_8_O_5_	P6	7428	
Flavonoids	Quercetin sulfate	C_15_H_8_O_11_S	P7	129639308	[16]
	Kaempferol sulfate	C_15_H_8_O_10_S	P8	129661100	
	Procyanidin B	C_30_H_26_O_12_	P9	122738	
	Prodelphinidin B	C_30_H_26_O_14_	P10	5089687	
	Quercetin 3-O-β-D-galactopyranoside (Hyperoside)	C_21_H_20_O_12_	P11	5281643	
	Quercetin 3-O-β-D-glucoside (Isoquercitrin)	C_21_H_20_O_12_	P12	5280804	
	Quercetin 3-O-β-D-glucuronide (Miquelianin)	C_21_H_18_O_13_	P13	5274585	
	Quercetin 3-O-α-L-arabinopyranoside (Guajavarin)	C_20_H_18_O_11_	P14	5481224	
	Quercetin 3-O-β-D-rhamnoside (Quercitrin)	C_21_H_20_O_11_	P15	5280459	
	(Epi)catechin gallate	C_22_H_18_O_10_	P16	107905	
	(−)-Epicatechin	C_15_H_14_O_6_	P17	72276	
	(+)-Catechin	C_15_H_14_O_6_	P18	9064	[15,16]
	Rutin ^2^	C_27_H_30_O_16_	P19	5280805	[15,17]
	Quercetin ^2^	C_15_H_10_O_7_	P20	5280343	
	Kaempferol	C_15_H_10_O_6_	P21	5280863	[15]
	Isorhamnetin	C_16_H_12_O_7_	P22	5281654	
Phenolic acid (Indole)	Tryptophan	C_11_H_12_N_2_O_2_	T1	6305	[17]
**Essential Oils**					
Essential Oils	Decanal	C_10_H_20_O	O1	8175	[18,19,20,31]
	Dodecanal	C_12_H_24_O	O2	8194	
	β-Caryophyllene	C_15_H_24_	O3	5281515	[18,19,20]
	Poligodial	C_15_H_22_O_2_	O4	72503	[32]
	Drimenol	C_15_H_26_O	O5	3080551	[18,19]
	Citral	C_10_H_16_O	O6	638011	[18]
	Euparone	C_12_H_10_O_4_	O7	104654	
	2,4-Heptadiene,2,6-dimethyl	C_9_H_16_	O8	5370124	
	*cis*-Caryophyllene	C_15_H_24_	O9	5281522	[31]
	*n*-Undecane	C_11_H_24_	O10	14257	
	Pentacosane	C_25_H_52_	O11	12406	[33]
	α-Curcumene	C_15_H_22_	O12	92139	[19]
	Eremophillene	C_15_H_24_	O13	6428416	
	7-Epi-α-selinene	C_15_H_24_	O14	10726905	
	Ledol	C_15_H_26_O	O15	92812	
	Nerolidol	C_15_H_26_O	O16	5284507	
	Caryophyllene oxide	C_15_H_24_O	O17	1742210	
	Eupatoriochromene	C_13_H_14_O_3_	O18	100768	
	α-Humulene	C_15_H_24_	O19	5281520	[19,31,33]
	1-Decanol	C_10_H_22_O	O20	8174	
	1-Dedocanol	C_12_H_26_O	O21	8193	

^1^ PubChem NID was accessed on https://pubchem.ncbi.nlm.nih.gov/ (accessed on 14 February 2026). ^2^ Phenolic acids and flavonoids were detected within the methanolic extract of *P. odorata* by our group.

**Table 3:** Types of interactions between phenolics and amino acids of the α-glucosidase’s active site.

Phenolics				
Codes	Δ*G* (kcal/mol)	*K*_i_ (μM)	H-Bonds (Å)	Hydrophobic Interactions
P1	−6.72	11.85	K156 (2.15); S241 (2.19); H280 (1.76); D307 (1.93); S311 (2.18, 2.50); R315 (2.77).	K156; R315.
P2	−5.13	173.10	K156 (1.76); Y158 (3.03); S241 (2.04); D242 (1.81).	K156; Y158; L177.
P3	−5.81	55.09	K156 (1.84); Y158 (2.89); S241 (1.94, 2.09); R315 (2.38).	K156.
P4	−4.65	391.23	K156 (2.02, 2.09, 2.82); S241 (2.39); D242 (1.98, 2.17).	K156.
P5	−4.76	326.24	Y158 (2.11); L313 (2.72); N415 (1.75).	F314.
P6	−5.52	89.65	K156 (2.07); S241 (2.33); D242 (1.79, 1.96, 3.03).	K156; Y158.
P7	−8.25	0.894	K156 (2.21); D307 (1.88, 1.88); R315 (2.88); E411 (2.62); N415 (1.97, 2.21).	R315; F314.
P8	−8.34	0.773	K156 (1.94); S241 (1.79); R315 (2.91); E411 (1.71); R442 (3.06).	Y158; F159; R315.
P9	−9.90	0.055	K156 (2.21, 2.21); Y158 (1.92, 3.04); S241 (1.82, 2.27); D242 (2.32); H280 (2.18); D307 (2.02); S311 (1.96); N415 (2.88).	K156; Y158; D242; R315.
P10	−6.00	40.14	D69 (1.72, 2.15); H112 (2.70); Y158 (2.13); D215 (2.02); D242 (2.07, 2.58); N350 (1.92); D352 (2.03); Q353 (3.04); E411 (2.86); R442 (1.97).	Y158; F178; D215; V216; F303; R315; R442.
P11	−8.15	1.06	D69 (3.21); Y158 (2.16); E277 (2.23, 3.00); Q279 (2.17); H280 (2.84); D307 (2.13, 2.21); R315 (2.02, 2.38, 2.82); D352 (1.90); Q353 (2.23).	Y158; F178; R315; D325; R442.
P12	−7.37	3.97	S157 (1.89, 2.73); Q279 (2.44); S311 (2.02, 2.20); E411 (2.39); N415 (1.79).	Y158; R315.
P13	−6.26	25.94	S157 (2.14); Q279 (2.44); S311 (1.98, 2.28); E411 (2.46); N415 (1.99).	Y158; R316.
P14	−7.62	2.60	T306 (2.83); D307 (1.96); S311 (2.13); R315 (1.96); Q353 (1.91, 2.07); N415 (2.12).	F303; R315.
P15	−8.16	1.04	E277 (1.96); Q279 (2.01); D307 (1.73, 1.99); R315 (2.58); Q353 (2.21); R442 (2.17).	Y158; F178; R315; R442.
P16	−8.79	0.359	D69 (1.71); E277 (1.77); T306 (2.38); D307 (2.23); Q353 (2.16, 2.52); R442 (2.41).	Y72; Y158; V216; F303; R315; D352; E411.
P17	−7.30	4.48	D69 (2.03); D215 (2.02); Q279 (2.77); R315 (2.39); Q353 (1.87); E411 (1.97); R442 (2.24).	F178; V216; F303; D352; R442.
P18	−7.24	4.95	D215 (2.03, 2.14); E277 (1.72); R315 (2.73); E411 (1.75); R442 (2.26).	Y72; D215; V216; F303.
P19	−9.42	0.125	D69 (1.80, 1.90); S157 (1.94); D215 (2.17); E277 (2.45); H280 (2.10); R315 (3.06, 3.08); D352 (2.74); E411 (1.89); N415 (1.81); R442 (2.79).	Y72; Y158; R315.
P20	−7.64	2.52	D69 (1.77); D215 (1.97); Q279 (2.07, 2.88); R315 (2.04, 2.56); H351 (2.55); D352 (1.90); R442 (2.29).	Y72; E411; R442.
P21	−7.26	4.80	D69 (1.77); H112 (2.79); Q279 (1.84); R315 (2.25); D352 (1.73); R442 (2.29).	Y72; V216; D353; E411.
P22	−7.07	3.54	D215 (1.99); Q279 (1.90); R315 (2.28); D352 (1.70); R442 (2.43).	D69; Y72; D215; V216; E277; E411.
T1	−6.57	15.22	E277 (1.79); H351 (1.96); R442 (2.10).	Y72; V216; D352.
Acarbose	−7.30	4.43	D69 (1.81, 1.87); Y158 (2.21); D215 (1.72); Q279 (2.31, 2.36); D307 (2.31, 3.05); P312 (2.47); R442 (2.53).	F303.

∆*G*: *Gibbs* binding energy score, *K*_i_: inhibition constant. The underlined amino acids are implicated in similar interactions observed with acarbose.

**Table 4:** Types of interactions between essential oils and amino acids of the α-glucosidase’s active site.

Essential Oils				
Codes	Δ*G* (kcal/mol)	*K*_i_ (µM)	H-Bonds (Å)	Hydrophobic Interactions
O1	−4.33	673.55	S241 (1.90).	K156; Y158; F314; R315.
O2	−4.14	928.11	R442 (2.58, 2.72); R446 (1.88).	Y72; F159; F178; V216.
O3	−6.74	11.38	-	K156; Y158.
O4	−6.94	8.11	S241 (1.76).	K156; Y158; F314.
O5	−6.72	11.96	R213 (2.14, 2.92); D352 (1.88).	Y72; Y158; F159; F178; V216; F303.
O6	−5.06	194.35	S241 (1.67).	K156; F314; Y316.
O7	−7.41	3.7	K156 (2.14); L313 (2.82); R315 (2.01).	K156; Y158.
O8	−4.51	493.36	-	K156; Y158; L177.
O9	−6.29	24.49	-	Y158; F159; F178; V216; F303.
O10	−4.17	876.17	-	K156; Y158; F314; R315; Y316.
O11	−5.17	162.55	-	Y72; K156; Y158; F178; V216; F314; R315.
O12	−6.23	27.2	-	K156; Y158; F314; R315; Y316.
O13	−6.56	15.49	-	K156; Y158; R315.
O14	−6.3	24.05	-	K156; Y158; R315.
O15	−6.55	15.81	N415 (2.20).	K156; F314; R315; Y316.
O16	−6.27	25.24	D69 (1.92); R442 (2.16).	Y72; Y158; F178; V216; F303.
O17	−6.54	16.15	R315 (2.11).	K156; Y158; R315.
O18	−6.32	23.46	E277 (2.10); Q279 (2.39); R442 (1.83).	Y158; F178; V216; F303; D352.
O19	−6.34	22.71		K156; Y158.
O20	−4.08	1010	D69 (1.75); R442 (2.01).	Y72; Y158; F159; F178; V216.
O21	−4.18	868.28	D69 (2.00); H112 (1.84).	Y72; F159; F178; V216.
Acarbose	−7.3	4.43	D69 (1.81, 1.87); Y158 (2.21); D215 (1.72); Q279 (2.31, 2.36); D307 (2.31, 3.05); P312 (2.47); R442 (2.53)	F303.

∆*G*: *Gibbs* binding energy score, *K*_i:_ inhibition constant. The underlined amino acids are implicated in similar interactions observed with acarbose.

**Table 5:** Drug-likeness and ADMET properties of P9, P19, and acarbose.

Pharmacokinetics	Parameters	P9	P19	Acarbose	Standard
Lipinski’s Ro5	Molecular weight	585.52	610.52	645.6	<500
	Rotatable bonds	3	6	9	<10
	H-bond acceptors	12	16	19	<10
	H-Bond donors	10	10	14	<5
	TPSA	220.76	269.43	321.17	<140
	MlogP	−0.26	−3.89	−6.94	<4.15
	Violations/Ro5	3/No	3/No	3/No	0/Yes
Absorption	Water solubility	−2.892	−2.892	−1.482	Log mol/L
	Caco2 permeability	−1.225	−0.949	−0.481	Log P_app_ in 10^−6^ cm/s
	HIA (%)	66.749	23.446	4.172	% Absorbed
	P-gp substate	Yes	Yes	Yes	Yes/No
	P-gp inhibitor I	Yes	No	No	Yes/No
	P-gp inhibitor II	Yes	No	No	Yes/No
Distributions	Human VDs	−0.158	1.663	−0.836	Log L/kg
	BBB permeability	−1.940	−1.899	−1.717	Log BB
Metabolism	CYP2D6 substrate	No	No	No	Yes/No
	CYP3A4 substrate	No	No	No	Yes/No
	CYP1A2 inhibitor	No	No	No	Yes/No
	CYP2C19 inhibitor	No	No	No	Yes/No
	CYP2C9 inhibitor	No	No	No	Yes/No
	CYP2D6 inhibitor	No	No	No	Yes/No
	CYP3A4 inhibitor	No	No	No	Yes/No
Excretion	Total clearance	−0.085	−0.369	0.428	Log mL/min/kg
	Renal OCT2 substrate	Yes	No	No	Yes/No
Toxicity	AMES toxicity	No	No	No	Yes/No
	MRTD	0.438	0.452	0.435	Log mg/kg/day
	hERG I/II inhibitors	No/Yes	No/Yes	No/Yes	Yes/No
	ORAT (LD_50_)	2.482	2.491	2.449	mol/kg
	Hepatotoxicity	No	No	No	Yes/No
	*T. Pyriformis* toxicity	0.285	0.285	0.285	Log μg/L

HIA: human intestinal absorption, P-gp: P-glycoprotein, VD: volume of distribution, BBB: brain–blood barrier, CYP: cytochrome P450, OCT2: organic cation transporter-2, MRTD: max. recommended tolerated dose, hERG: human ether-a-go-go-related gene, ORAT: oral rate acute toxicity, and LD_50_: half-lethal dose. P9: procyanidin B, and P19: rutin.

## Data Availability

The original contributions presented in this study are included in the article/Supplementary Material. Further inquiries can be directed to the corresponding author.

## Supplementary Materials

The following supporting information can be downloaded at https://www.mdpi.com/article/10.3390/ijms27156885/s1.

## Abbreviations

The following abbreviations are used in this manuscript:

ADMET	Absorption, Distribution, Metabolism, Excretion, and Toxicity
BBB	Brain–Blood Barrier
E_0_	Free Enzyme
ES	Enzyme–Substrate
H-bond	Hydrogen Bond
HIA	Human Intestinal Absorption
*K*_i_	Inhibition Constant
*K*_m_	Michaelis Constant
MD	Molecular Dynamics
PDB	Protein Data Bank
P-gp	P-glycoprotein
RMSD	Root Mean Square Deviation
TPSA	Topological Polar Surface Area
VD	Volume Distribution
V_max_	Maximum Velocity
[S]	Substrate
[V]	Velocity
[V_0_]	Initial Velocity
