# Supplementary material for: Exploring the α-Glucosidase Inhibitory Activity of Bioactive Compounds from Persicaria odorata (Lour.) Extracts via Integrated In Vitro and In Silico Studies
Source: Int J Mol Sci. 2026 Aug 1;27(15):6885. doi: 10.3390/ijms27156885 (PMC13467365; doi:10.3390/ijms27156885)
Supplement: Supplementary file 1 [file ijms-27-06885-s001.zip › ijms-4432748-supplementary/Supplementary Materials 1 Figures S1-S2.pdf]

Supplementary Material 1: Chemical Structures of Phenolics and Essential Oils

1. Phenolics

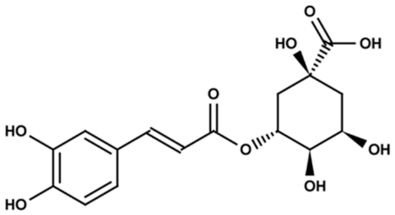

(P1)

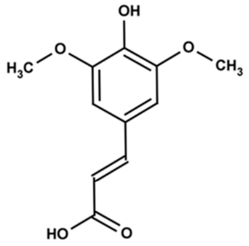

(P2)

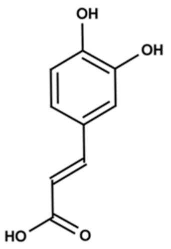

(P3)

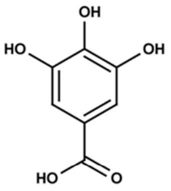

(P4)

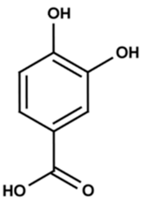

(P5)

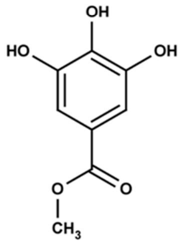

(P6)

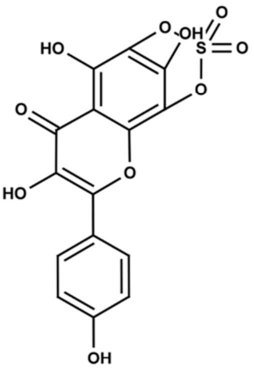

(P8)

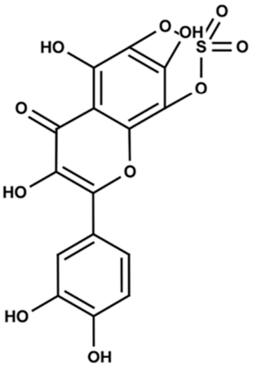

(P7)

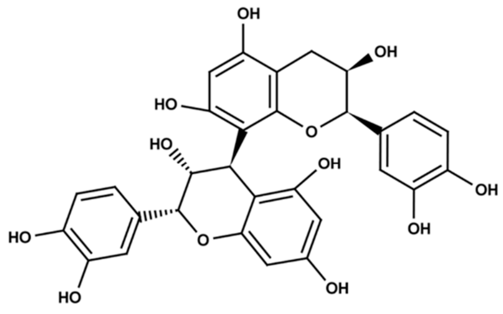

(P9)

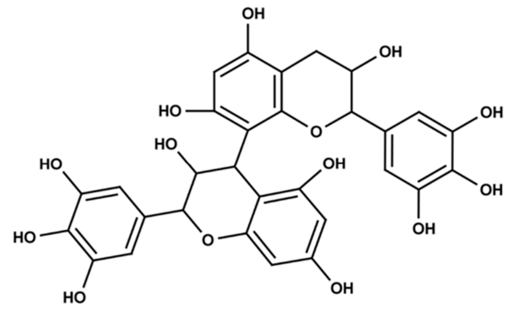

(P10)

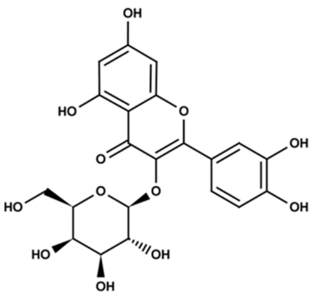

(P11)

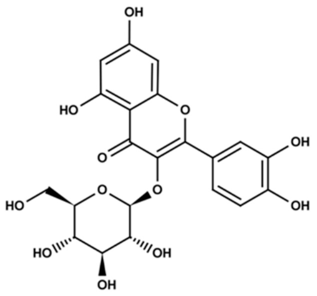

(P12)

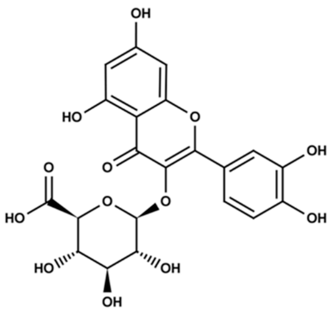

(P13)

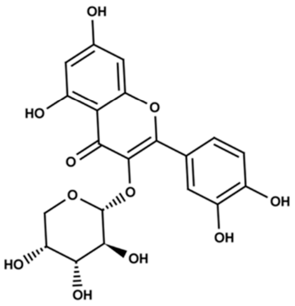

(P14)

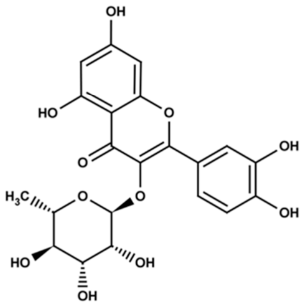

(P15)

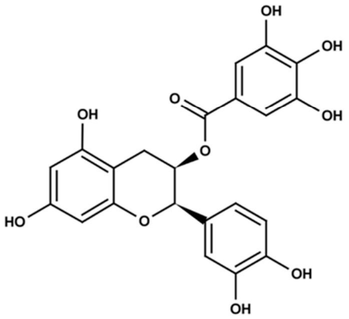

(P16)

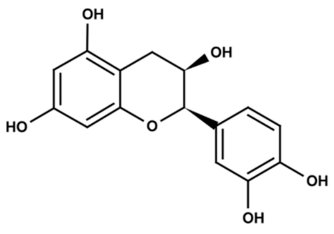

(P17)

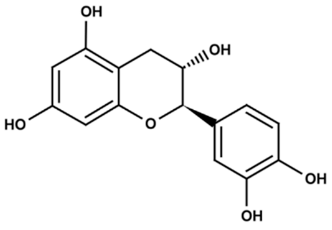

(P18)

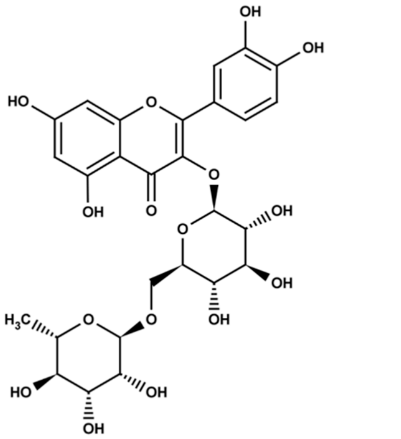

(P19)

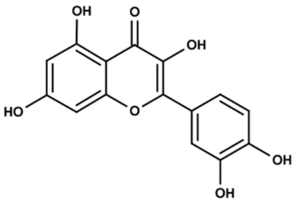

(P20)

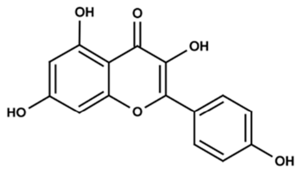

(P21)

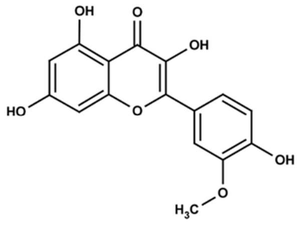

(P22)

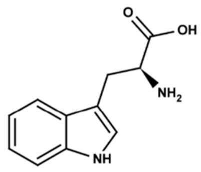

(T1)

Figure S1. Chemical structures of phenolics reported in *P. odorata* extracts.

2. Essential Oils

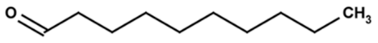

(O1)

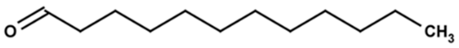

(O2)

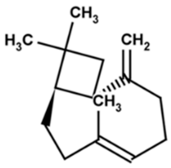

(O3)

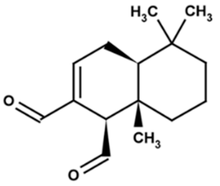

(O4)

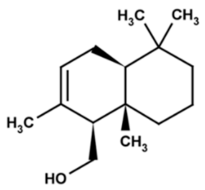

(O5)

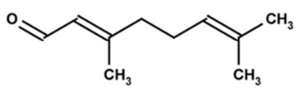

(O6)

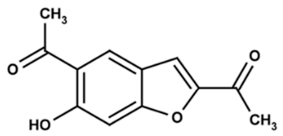

(O7)

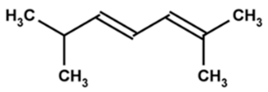

(O8)

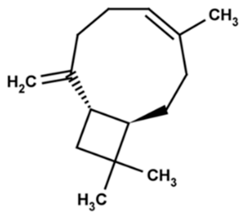

(O9)

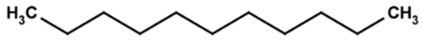

(O10)

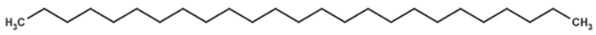

(O11)

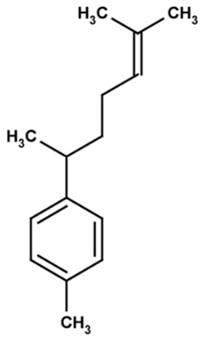

(O12)

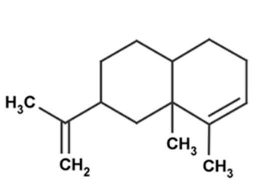

(O13)

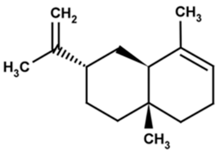

(O14)

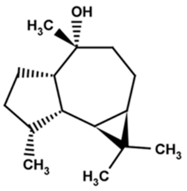

(O15)

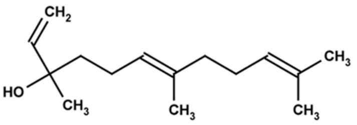

(O16)

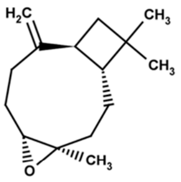

(O17)

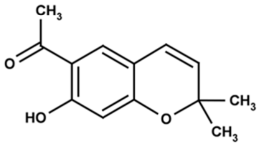

(O18)

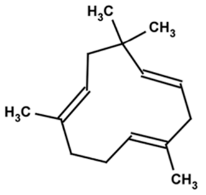

(O19)

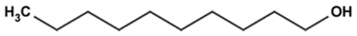

(O20)

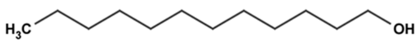

(O21)

Figure S2. Chemical structures of essential oils reported in *P. odorata* extracts.
