# Supplementary material for: Exploring the α-Glucosidase Inhibitory Activity of Bioactive Compounds from Persicaria odorata (Lour.) Extracts via Integrated In Vitro and In Silico Studies
Source: Int J Mol Sci. 2026 Aug 1;27(15):6885. doi: 10.3390/ijms27156885 (PMC13467365; doi:10.3390/ijms27156885)
Supplement: Supplementary file 1 [file ijms-27-06885-s001.zip › ijms-4432748-supplementary/Supplementary Materials 2 Figures S3-S5.pdf]

Supplementary Material 2: Chromatograms of Each Compound by HPLC-DAD Analysis

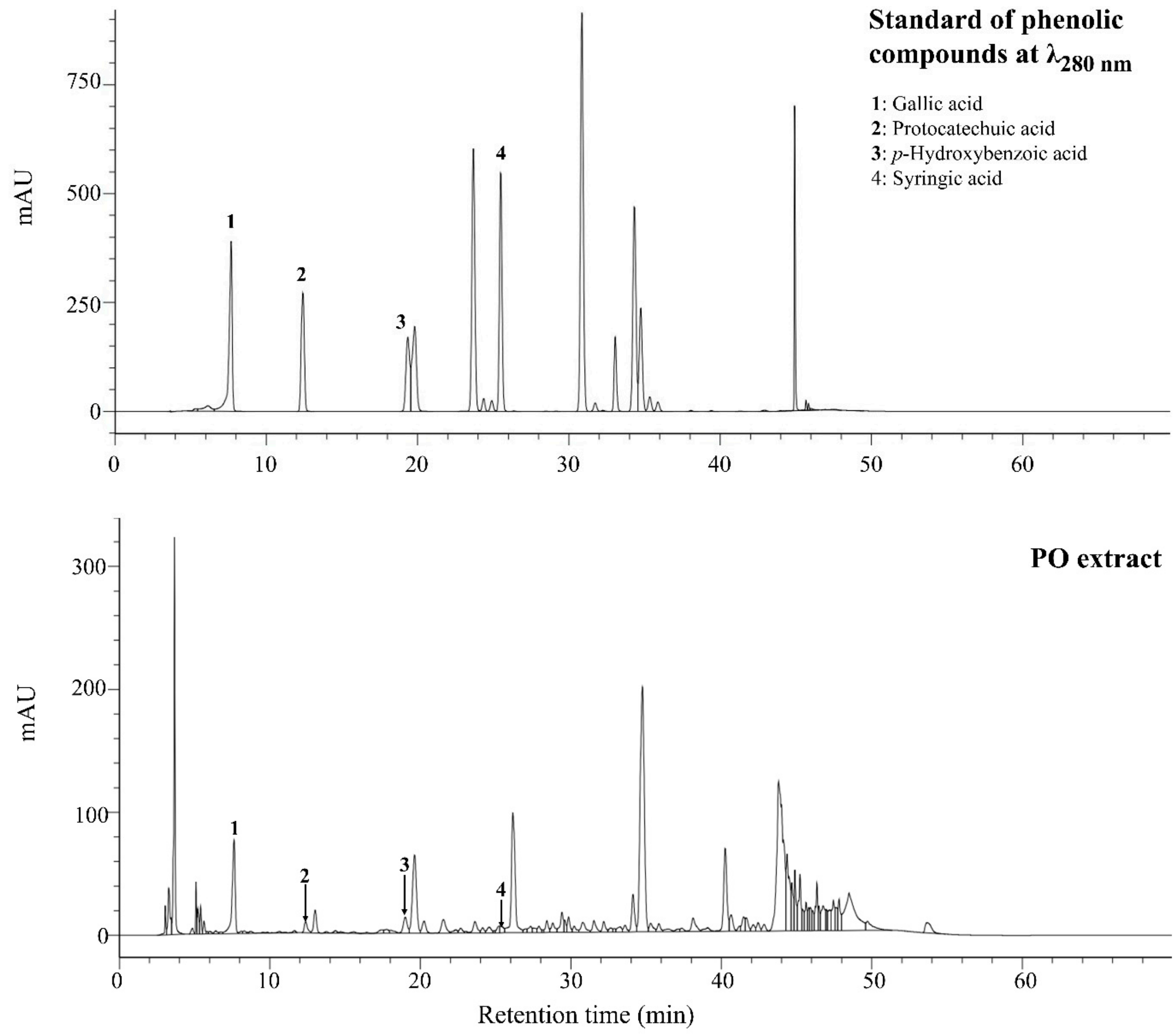

Figure S3. Chromatogram of phenolic compound standards and the methanolic extracts of *P. odorata* at 280 nm

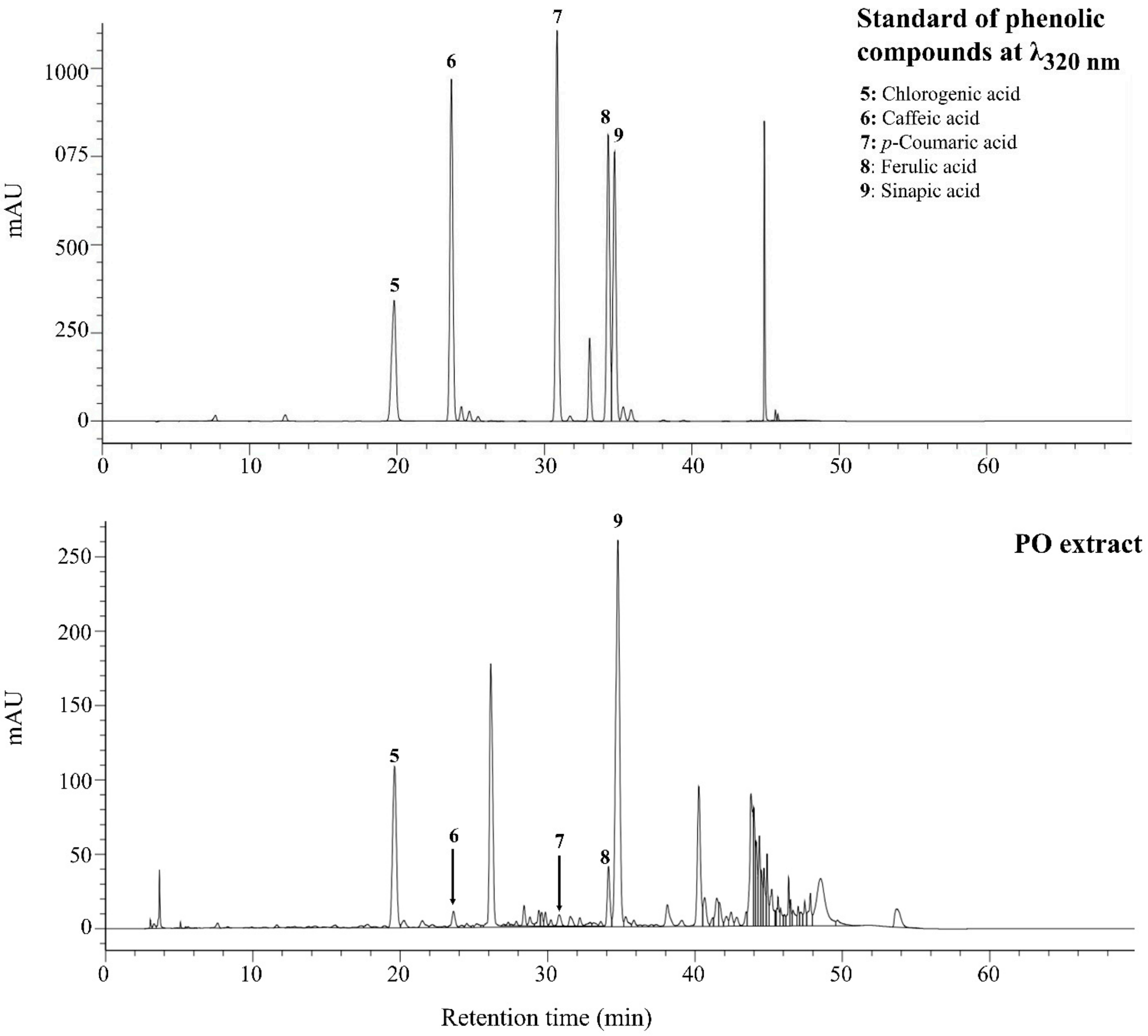

Figure S4. Chromatogram of phenolic compound standards and the methanolic extracts of *P. odorata* at 320 nm

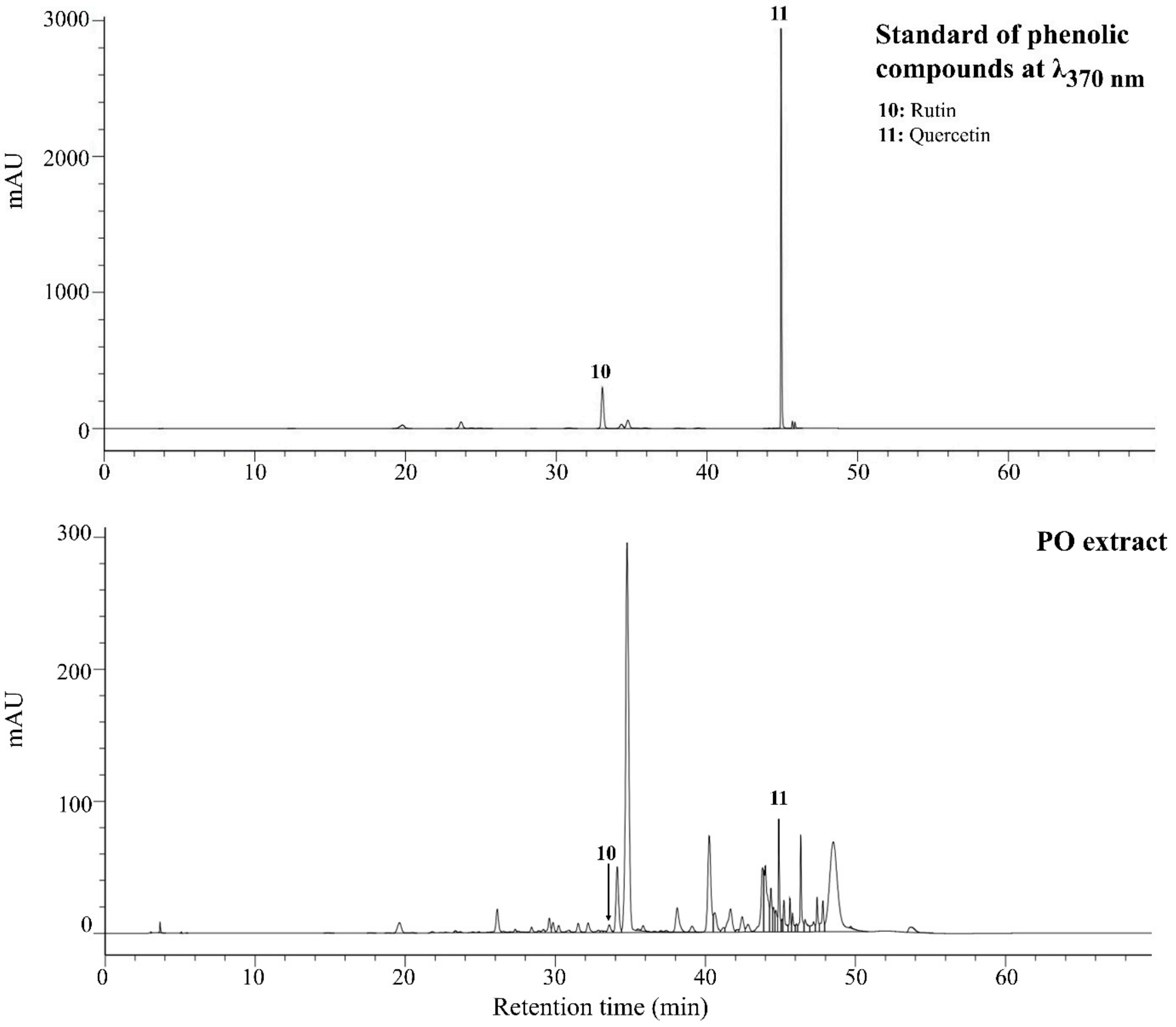

Figure S5. Chromatogram of phenolic compound standards and the methanolic extracts of *P. odorata* at 370 nm
