# Supplementary material for: Exploring the α-Glucosidase Inhibitory Activity of Bioactive Compounds from Persicaria odorata (Lour.) Extracts via Integrated In Vitro and In Silico Studies
Source: Int J Mol Sci. 2026 Aug 1;27(15):6885. doi: 10.3390/ijms27156885 (PMC13467365; doi:10.3390/ijms27156885)
Supplement: Supplementary file 1 [file ijms-27-06885-s001.zip › ijms-4432748-supplementary/Supplementary Materials 3 Figures S6-S7.pdf]

Supplementary Material 3: Molecular Docking Interactions (2D visualization)

1. Phenolics

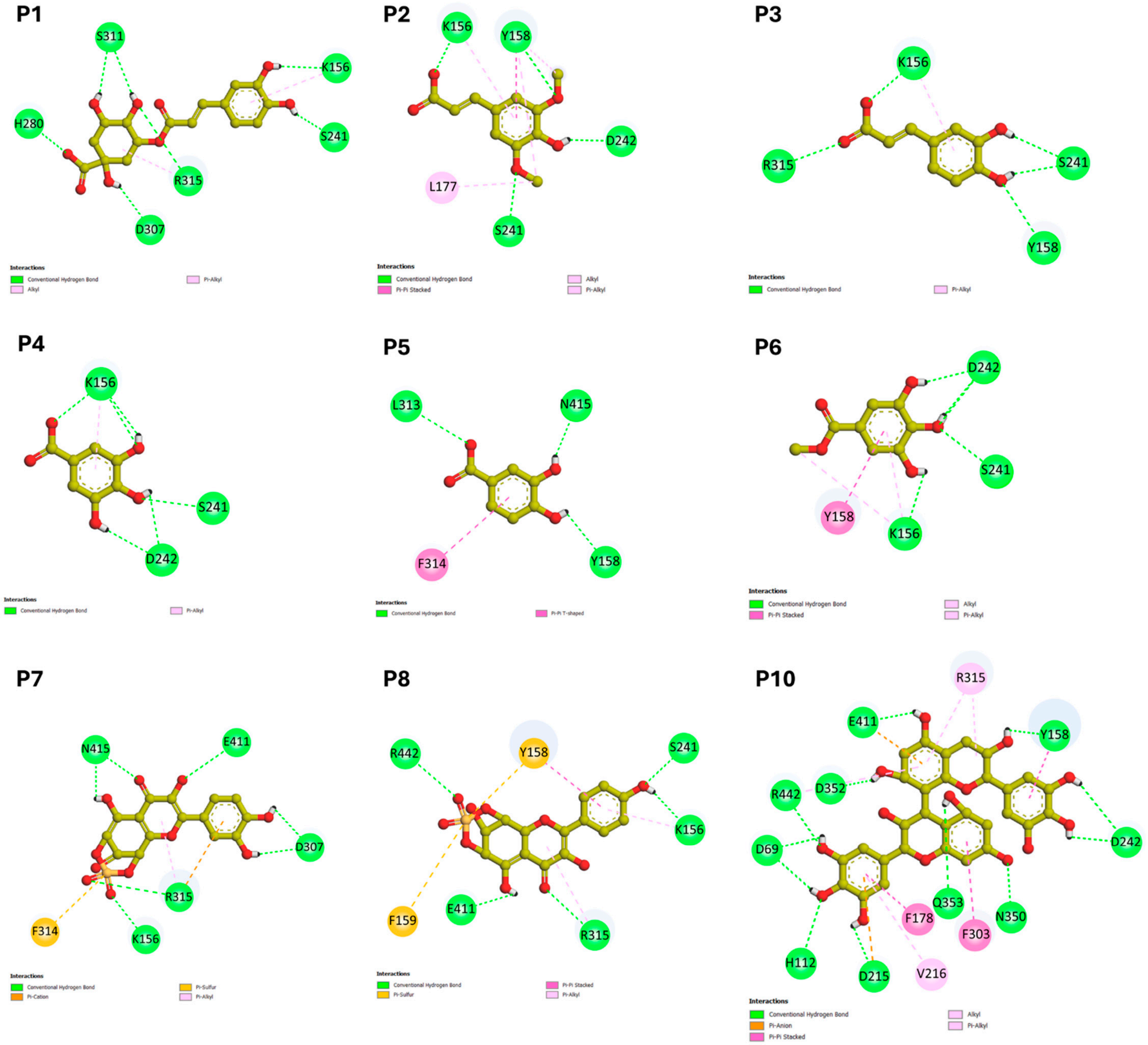

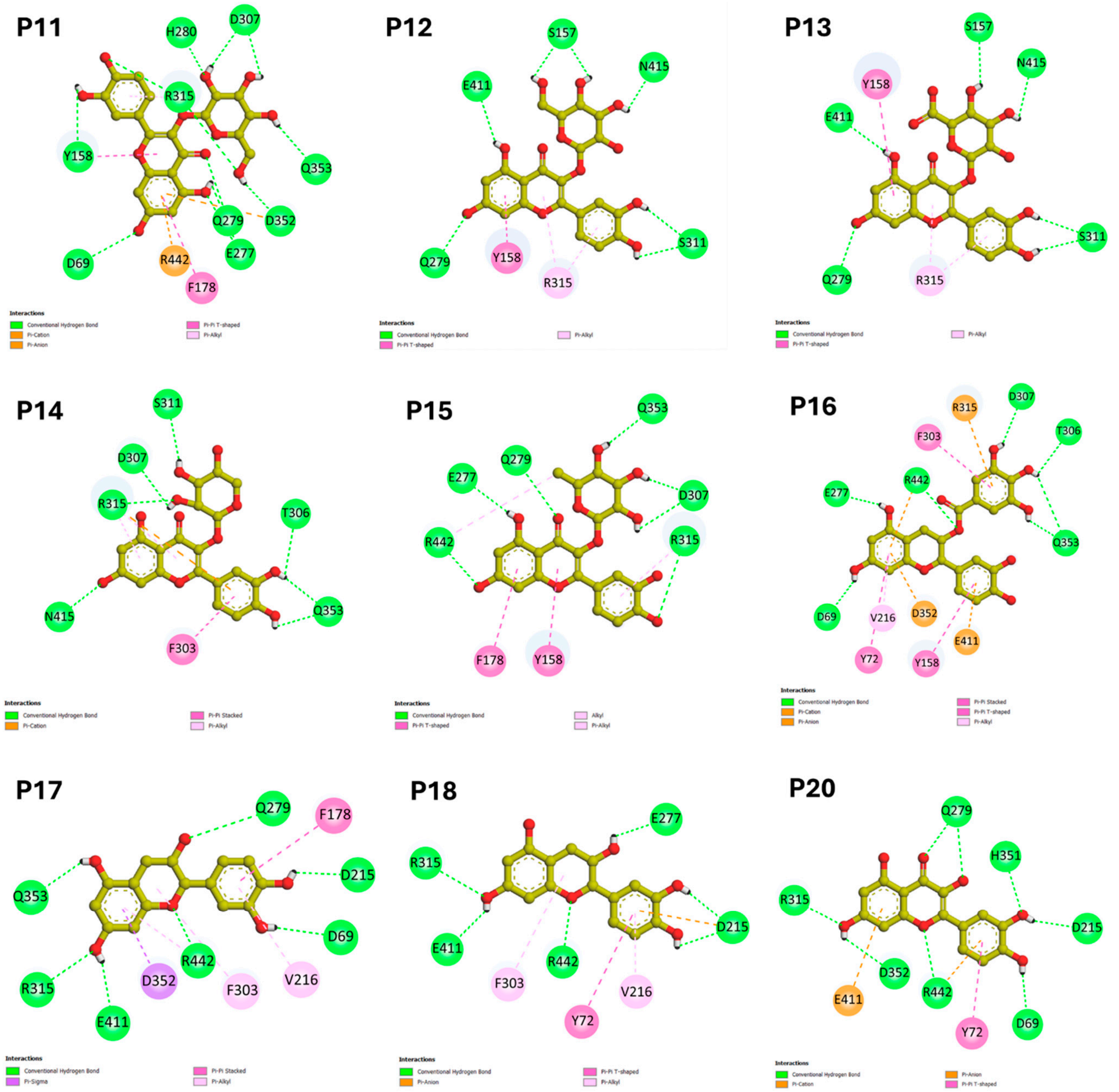
P11
H280
D307
R315
Y158
Q353
Q279
D352
E277
D69
R442
F178
Interactions
Conventional Hydrogen Bond
Pi-Cation
Pi-Anion
Pi-Pi T-shaped
Pi-Alkyl
P12
S157
E411
N415
S311
Q279
Y158
R315
Interactions
Conventional Hydrogen Bond
Pi-Pi T-shaped
Pi-Alkyl
P13
S157
Y158
N415
E411
S311
Q279
R315
Interactions
Conventional Hydrogen Bond
Pi-Pi T-shaped
Pi-Alkyl
P14
S311
D307
R315
T306
Q353
N415
F303
Interactions
Conventional Hydrogen Bond
Pi-Cation
Pi-Pi Stacked
Pi-Alkyl
P15
Q353
Q279
E277
D307
R442
R315
F178
Y158
Interactions
Conventional Hydrogen Bond
Pi-Pi T-shaped
Alkyl
Pi-Alkyl
P16
R315
D307
F303
T306
R442
E277
Q353
D69
V216
D352
E411
Y72
Y158
Interactions
Conventional Hydrogen Bond
Pi-Cation
Pi-Anion
Pi-Pi Stacked
Pi-Pi T-shaped
Pi-Alkyl
P17
Q279
F178
D215
Q353
D69
R442
D352
F303
V216
R315
E411
Interactions
Conventional Hydrogen Bond
Pi-Sigma
Pi-Pi Stacked
Pi-Alkyl
P18
E277
R315
D215
E411
R442
F303
V216
Y72
Interactions
Conventional Hydrogen Bond
Pi-Anion
Pi-Pi T-shaped
Pi-Alkyl
P20
Q279
H351
R315
D215
D352
R442
E411
D69
Y72
Interactions
Conventional Hydrogen Bond
Pi-Cation
Pi-Anion
Pi-Pi T-shaped

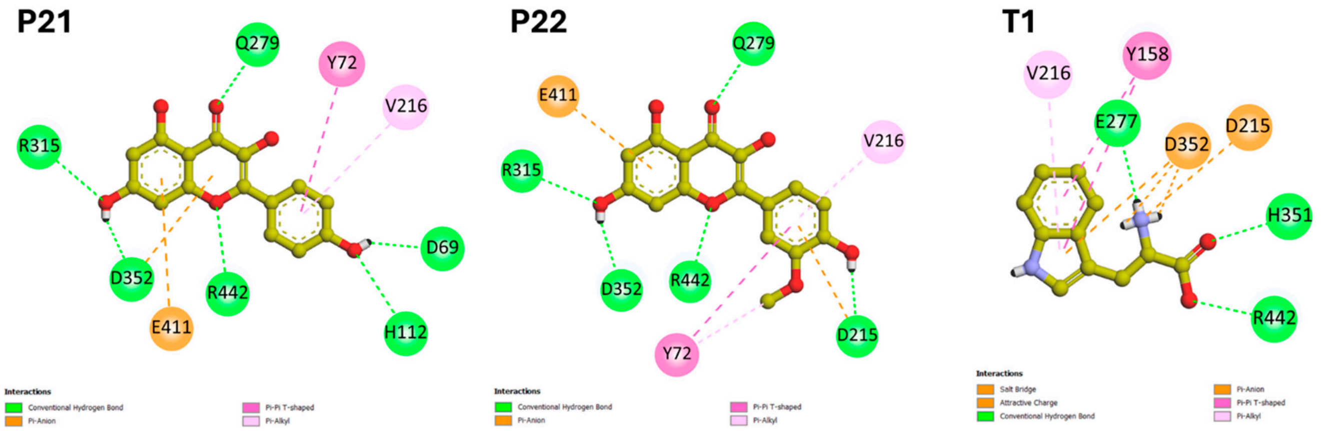

Figure S6. 2D visualization of molecular interactions between phenolics and amino acids of the active site of α-glucosidase (PDB ID: 3A4A).

2. Essential Oils

O1

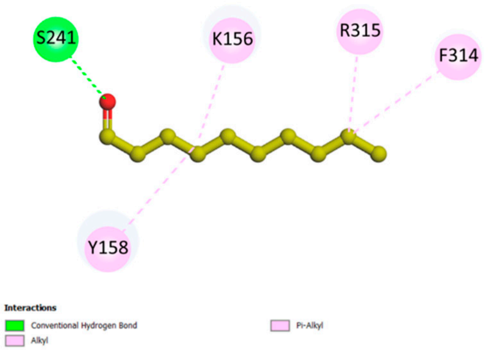

O2

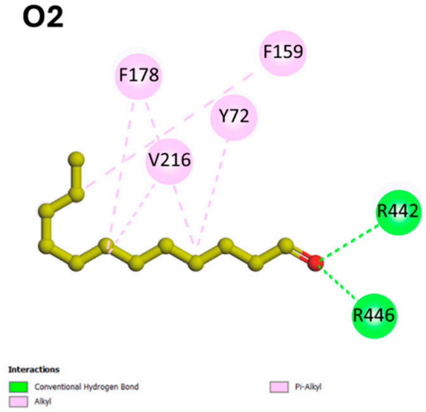

O3

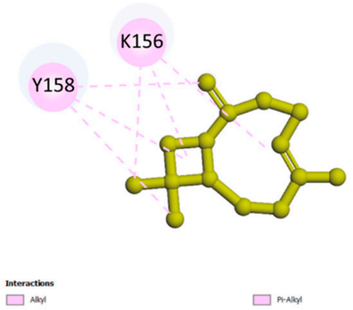

O4

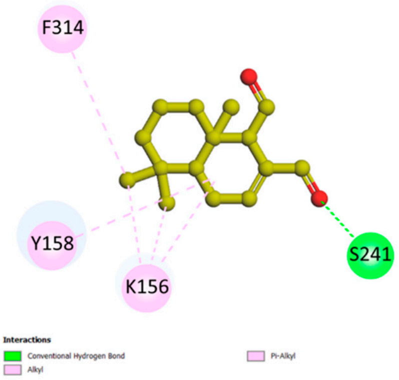

O5

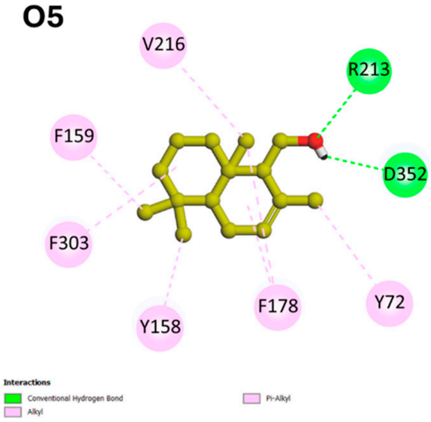

O6

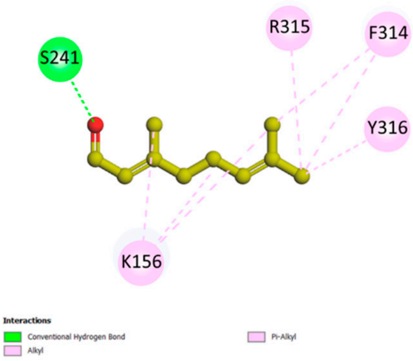

O7

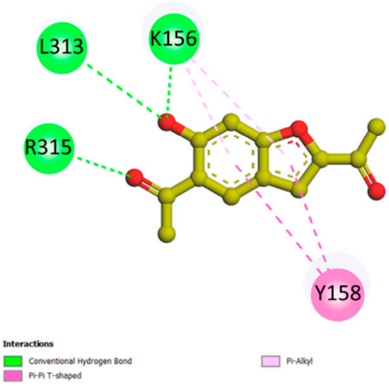

O8

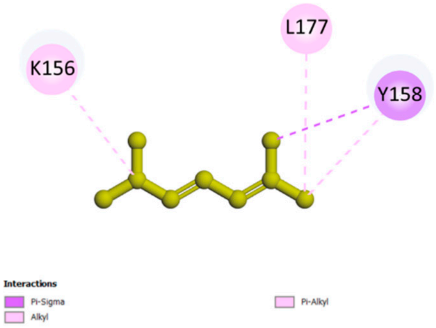

O9

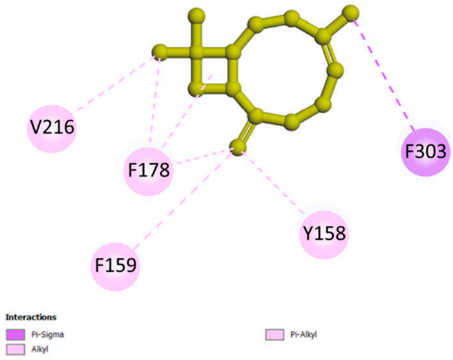

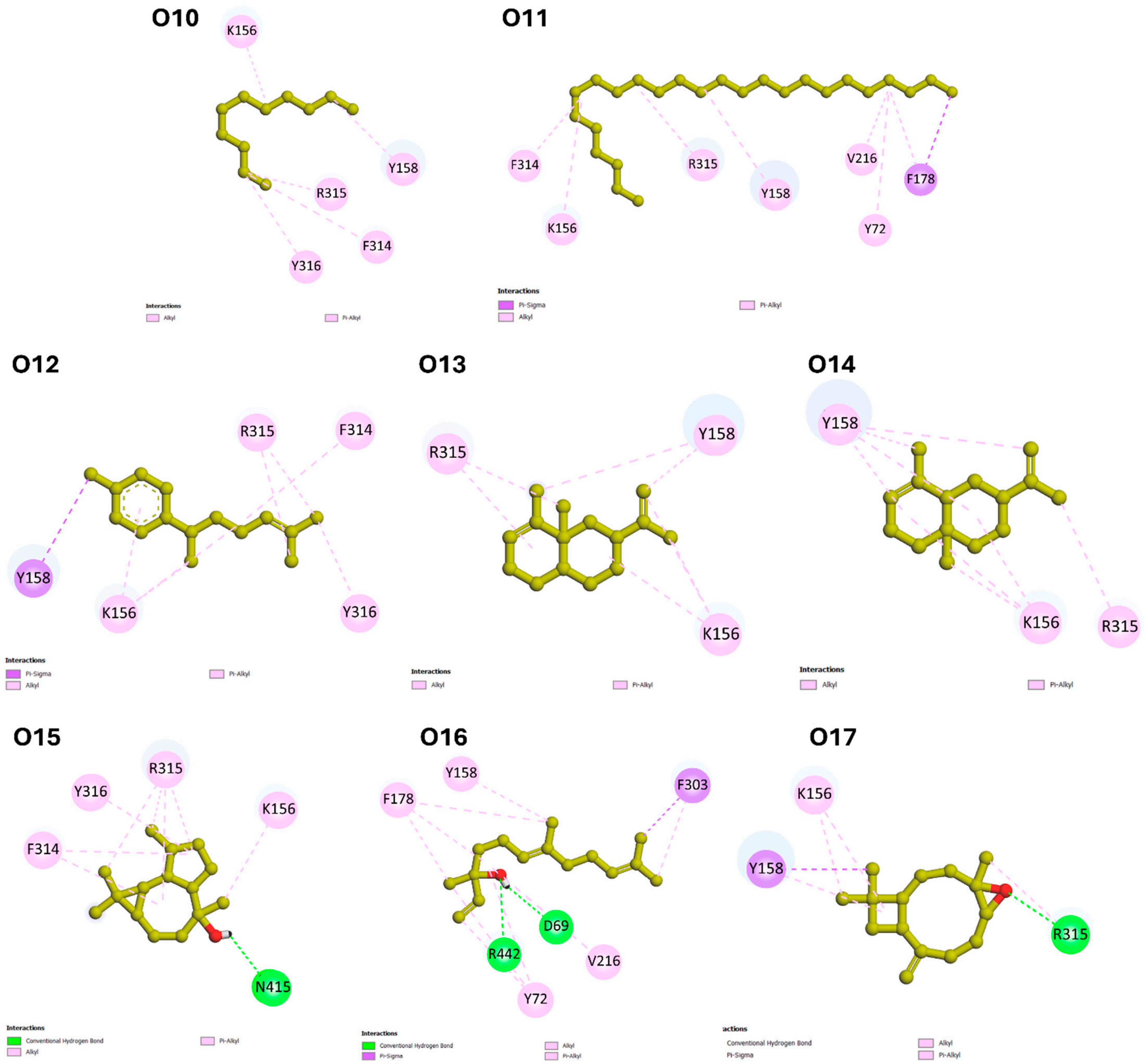
O10
K156
Y158
R315
F314
Y316
Interactions
Alkyl
Pi-Alkyl
O11
F314
R315
Y158
V216
F178
K156
Y72
Interactions
Pi-Sigma
Alkyl
Pi-Alkyl
O12
R315
F314
Y158
K156
Y316
Interactions
Pi-Sigma
Alkyl
Pi-Alkyl
O13
Y158
R315
K156
Interactions
Alkyl
Pi-Alkyl
O14
Y158
K156
R315
Interactions
Alkyl
Pi-Alkyl
O15
R315
Y316
K156
F314
N415
Interactions
Conventional Hydrogen Bond
Alkyl
Pi-Alkyl
O16
Y158
F178
F303
D69
R442
V216
Y72
Interactions
Conventional Hydrogen Bond
Pi-Sigma
Alkyl
Pi-Alkyl
O17
K156
Y158
R315
actions
Conventional Hydrogen Bond
Pi-Sigma
Alkyl
Pi-Alkyl

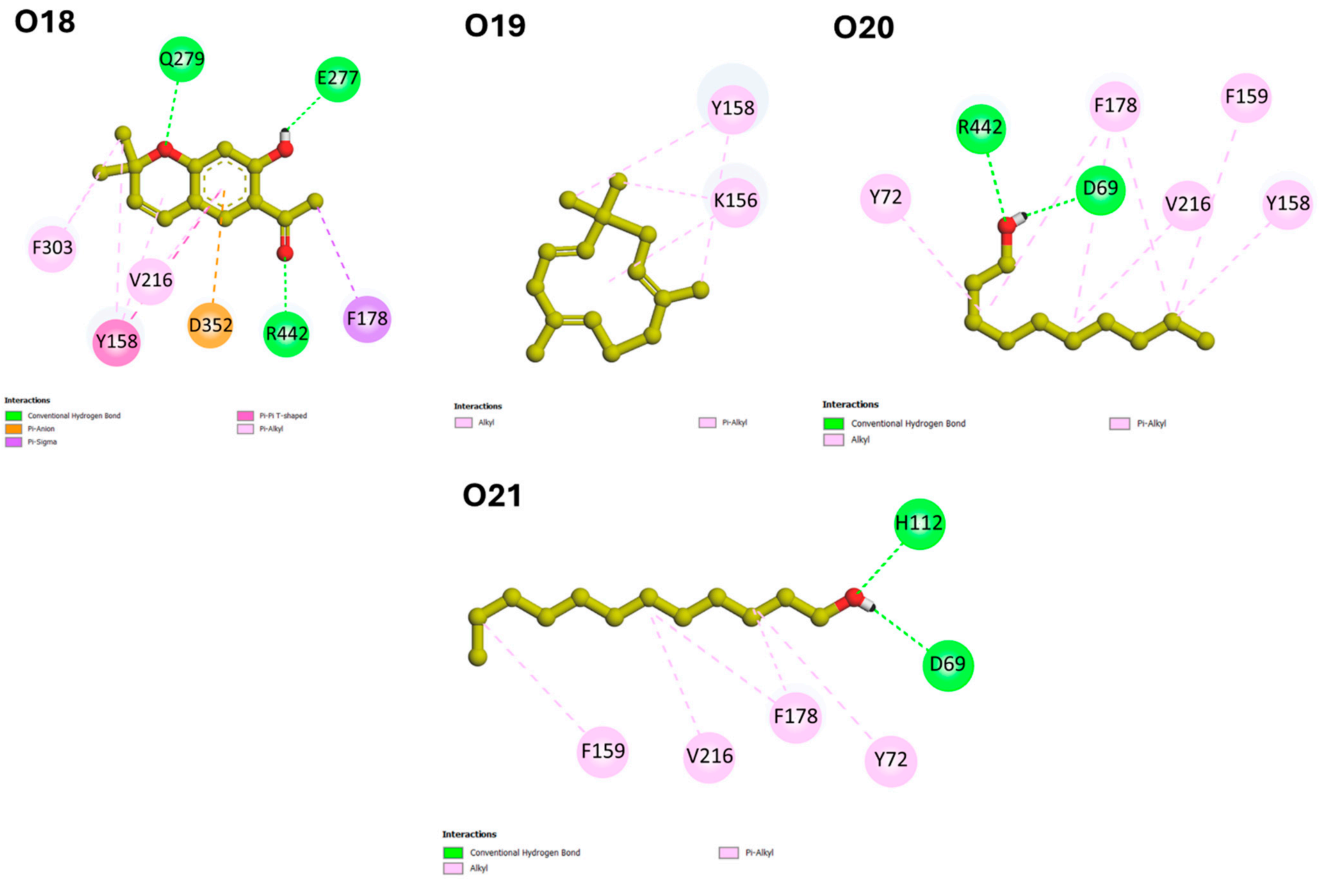

Figure S7. 2D visualization of molecular interactions between essential oils and amino acids of the active site of α-glucosidase (PDB ID: 3A4A).
