# Supplementary material for: Exploring the α-Glucosidase Inhibitory Activity of Bioactive Compounds from Persicaria odorata (Lour.) Extracts via Integrated In Vitro and In Silico Studies
Source: Int J Mol Sci. 2026 Aug 1;27(15):6885. doi: 10.3390/ijms27156885 (PMC13467365; doi:10.3390/ijms27156885)
Supplement: Supplementary file 1 [file ijms-27-06885-s001.zip › ijms-4432748-supplementary/Supplementary Materials 4 Tables S1-S6.pdf]

Supplementary Material 4: Drug-likeness and ADMET Properties

1. Drug-Likeness Properties by using SwissADME

Table S1. Drug-likeness properties of phenolics and essential oils identified in *P. odorata* extracts using SwissADME.

Codes of Compounds	MW	Rotatable Bonds	H-bond Acceptors	H-bond Donors	TPSA	MLOGP	Lipinski (Violations)	Lipinski's Rule
**A. Phenolics**								
P1	354.31	5	9	6	164.75	-1.05	1	No
P2	224.21	4	5	2	75.99	0.73	0	Yes
P3	180.16	2	4	3	77.76	0.70	0	Yes
P4	170.12	1	5	4	97.99	-0.16	0	Yes
P5	154.12	1	4	3	77.76	0.40	0	Yes
P6	184.15	2	5	3	86.99	0.18	0	Yes
P7	396.28	1	11	5	192.34	-1.23	1	No
P8	380.28	1	10	4	172.11	-0.74	0	Yes
P9	578.52	3	12	10	220.76	-0.26	3	No
P10	610.52	3	14	12	261.22	-1.22	3	No
P11	464.38	4	12	8	210.51	-2.59	2	No
P12	464.38	4	12	8	210.51	-2.59	2	No
P13	478.36	4	13	8	227.58	-2.60	2	No
P14	434.35	3	11	7	190.28	-2.06	2	No
P15	448.38	3	11	7	190.28	-1.84	2	No
P16	442.37	4	10	7	177.14	0.05	1	No
P17	290.27	1	6	5	110.38	0.24	0	Yes
P18	290.27	1	6	5	110.38	0.24	0	Yes
P19	610.52	6	16	10	269.43	-3.89	3	No
P20	302.24	1	7	5	131.36	-0.56	0	Yes
P21	286.24	1	6	4	111.13	-0.03	0	Yes
P22	316.26	2	7	4	120.36	-0.31	0	Yes
T1	204.23	3	3	3	79.11	-1.66	0	Yes
**B. Essential Oils**								
O1	156.27	8	1	0	17.07	2.70	0	Yes
O2	184.32	10	1	0	17.07	3.27	0	Yes
O3	204.35	0	0	0	0	4.63	1	No
O4	234.33	2	2	0	34.14	2.54	0	Yes
O5	222.37	1	1	1	20.23	3.67	0	Yes
O6	152.23	4	1	0	17.07	2.49	0	Yes
O7	218.21	2	4	1	67.51	0.21	0	Yes
O8	124.22	2	0	0	0	3.36	0	Yes
O9	204.35	0	0	0	0	4.63	1	No
O10	156.31	8	0	0	0	5.11	1	No
O11	352.68	22	0	0	0	8.45	1	No
O12	202.34	4	0	0	0	5.75	1	No
O13	204.35	1	0	0	0	4.63	1	No
O14	204.35	1	0	0	0	4.63	1	No
O15	222.37	0	1	1	20.23	3.81	0	Yes
O16	222.37	7	1	1	20.23	3.86	0	Yes

O17	220.35	0	1	0	12.53	3.67	0	Yes
O18	218.25	1	3	1	46.53	1.50	0	Yes
O19	204.35	0	0	0	0	4.53	1	No
O20	158.28	8	1	1	20.23	2.84	0	Yes
O21	186.33	10	1	1	20.23	3.41	0	Yes
Acarbose	645.60	9	19	14	321.17	-7.69	4	No

MW: molecular weight (≤ 500), rotatable bonds (≤ 10), TPSA: topological polar surface area (≤ 140), MlogP (≤ 4.15), H-bond donors (≤ 5), H-bond acceptors (≤ 10).

2. ADMET Properties by using pkCSM

Table S2. Absorption of phenolics and essential oils identified in *P. odorata* extracts.

Absorption						
**Codes of Compounds**	**Water Solubility (log mol/L)**	**Caco2 Permeability (log Papp in 10^{-6} cm/s)**	**HIA (%)**	**P-gp Substrate (Yes/No)**	**P-gp I Inhibitor (Yes/No)**	**P-gp II Inhibitor (Yes/No)**
**A. Phenolics**						
P1	-2.449	-0.840	36.377	Yes	No	No
P2	-2.869	0.272	93.064	Yes	No	No
P3	-2.330	0.634	69.407	No	No	No
P4	-2.560	-0.081	43.374	No	No	No
P5	-2.069	0.490	71.174	No	No	No
P6	-2.000	-0.056	76.635	Yes	No	No
P7	-3.106	0.343	69.450	Yes	No	No
P8	-3.336	0.383	66.784	Yes	No	No
P9	-2.892	-1.559	40.318	Yes	Yes	Yes
P10	-2.892	-1.225	66.749	Yes	Yes	Yes
P11	-2.971	-0.754	42.838	Yes	No	No
P12	-2.971	-0.754	42.838	Yes	No	No
P13	-2.956	-1.021	36.495	Yes	No	No
P14	-3.076	0.126	61.18	Yes	No	No
P15	-3.087	0.121	62.005	Yes	No	No
P16	-2.911	-1.264	62.096	Yes	No	Yes
P17	-3.117	-0.283	68.829	Yes	No	No
P18	-3.117	-0.283	68.829	Yes	No	No
P19	-2.892	-0.949	23.446	Yes	No	No
P20	-2.925	-0.229	77.207	Yes	No	No
P21	-3.04	0.032	74.29	Yes	No	No
P22	-3.000	-0.003	76.014	Yes	No	No
T1	-2.891	0.638	77.224	Yes	No	No
**B. Essential Oils**						
O1	-4.337	1.484	94.394	No	No	No
O2	-5.499	1.482	93.707	No	No	No
O3	-5.555	1.423	94.845	No	No	No
O4	-3.975	1.625	97.839	No	Yes	No
O5	-4.505	1.498	92.75	No	No	No
O6	-3.377	1.504	95.317	No	No	No
O7	-2.803	0.973	92.787	No	No	No

O8	-4.286	1.413	94.557	No	No	No
O9	-5.555	1.423	94.845	No	No	No
O10	-6.156	1.379	92.764	No	No	No
O11	-7.936	1.128	87.953	No	No	Yes
O12	-5.962	1.537	93.29	No	No	No
O13	-6.074	1.401	94.127	No	No	No
O14	-6.074	1.401	94.127	No	No	No
O15	-4.313	1.483	92.814	No	No	No
O16	-5.176	1.498	91.887	No	No	No
O17	-4.321	1.414	95.669	No	No	No
O18	-3.083	1.297	93.134	No	No	No
O19	-5.191	1.421	94.682	Yes	No	No
O20	-3.829	1.469	91.865	No	No	No
O21	-5.017	1.468	91.178	No	No	No

Caco2 permeability: ≥ 0.9 cm/s (high), HIA: human intestinal absorption: ≤ 30% (poorly absorbed), P-gp: P-glycoprotein.

Table S3. Distribution of phenolics and essential oils identified in *P. odorata* extracts.

Distribution					
**Codes of Phenolics**	**Human VDs (log L/kg)**	**BBB Permeability (Log BB)**	**Codes of Essential Oils**	**Human VDs (log L/kg)**	**BBB permeability (Log BB)**
P1	0.581	-1.407	O1	0.299	0.705
P2	-1.110	-0.247	O2	0.393	0.743
P3	-1.098	-0.647	O3	0.652	0.733
P4	-1.855	-1.102	O4	0.300	0.333
P5	-1.298	-0.683	O5	0.472	0.610
P6	0.355	-1.046	O6	0.166	0.626
P7	0.910	-1.752	O7	0.187	-0.048
P8	0.494	-1.570	O8	0.284	0.758
P9	-0.158	-1.940	O9	0.652	0.733
P10	-0.005	-2.584	O10	0.537	0.844
P11	0.672	-1.755	O11	0.374	1.109
P12	0.672	-1.755	O12	1.089	0.593
P13	0.622	-1.855	O13	0.686	0.776
P14	0.436	-1.668	O14	0.686	0.776
P15	0.451	-1.690	O15	0.556	0.632
P18	0.664	-1.847	O16	0.370	0.652
P17	1.027	-1.054	O17	0.564	0.647
P16	1.027	-1.054	O18	0.053	0.135
P19	1.663	-1.899	O19	0.505	0.663
P20	1.559	-1.098	O20	0.299	0.685
P21	1.274	-0.939	O21	0.390	0.723
P22	1.123	-1.135	T1	-0.081	-0.495

VD: volume of distribution (log VD < 0.15 = low; log VD > 0.45 = high), BBB: blood-brain barrier (log-BB > -0.3 = readily cross, log-BB < -1 = poorly).

Table S4. Metabolism of phenolics and essential oils identified in *P. odorata* extracts.

**Metabolism**

Codes of Compounds	CYP2D6 substrate (Yes/No)	CYP3A4 substrate (Yes/No)	CYP1A2 inhibitor (Yes/No)	CYP2C19 inhibitor (Yes/No)	CYP2C9 inhibitor (Yes/No)	CYP2D6 inhibitor (Yes/No)	CYP3A4 inhibitor (Yes/No)
**A. Phenolics**							
P1	No	No	No	No	No	No	No
P2	No	No	No	No	No	No	No
P3	No	No	No	No	No	No	No
P4	No	No	No	No	No	No	No
P5	No	No	No	No	No	No	No
P6	No	No	No	No	No	No	No
P7	No	No	No	No	No	No	No
P8	No	No	No	No	No	No	No
P9	No	No	No	No	No	No	No
P10	No	No	No	No	No	No	No
P11	No	No	No	No	No	No	No
P12	No	No	No	No	No	No	No
P13	No	No	No	No	No	No	No
P14	No	No	No	No	No	No	No
P15	No	No	No	No	No	No	No
P16	No	No	No	No	No	No	No
P17	No	No	No	No	No	No	No
P18	No	No	No	No	No	No	No
P19	No	No	No	No	No	No	No
P20	No	No	Yes	No	No	No	No
P21	No	No	Yes	No	No	No	No
P22	No	No	Yes	No	No	No	No
T1	No	No	No	No	No	No	No
**B. Essential Oils**							
O1	No	No	No	No	No	No	No
O2	No	No	No	No	No	No	No
O3	No	No	No	No	No	No	No
O4	No	Yes	No	No	No	No	No
O5	No	Yes	No	No	No	No	No
O6	No	No	No	No	No	No	No
O7	No	No	Yes	No	No	No	No
O8	No	No	No	No	No	No	No
O9	No	No	No	No	No	No	No
O10	No	No	No	No	No	No	No
O11	No	Yes	Yes	No	No	No	No
O12	No	Yes	Yes	No	No	No	No
O13	No	Yes	No	No	No	No	No
O14	No	Yes	No	No	No	No	No
O15	No	Yes	Yes	No	No	No	No
O16	No	No	No	No	No	No	No
O17	No	No	Yes	Yes	Yes	No	No
O18	No	No	Yes	No	No	No	No
O19	No	No	No	No	No	No	No
O20	No	No	No	No	No	No	No

O21	No	No	No	No	No	No	No

Table S5. Excretion of phenolics and essential oils identified in *P. odorata* extracts.

Excretion					
**Codes of Phenolics**	**Total Clearance (log ml/min/kg)**	**Renal OCT2 Substrate (Yes/No)**	**Code of Essential Oils**	**Total Clearance (log ml/min/kg)**	**Renal OCT2 Substrate (Yes/No)**
P1	0.307	No	O1	1.608	No
P2	0.718	No	O2	1.682	No
P3	0.508	No	O3	1.088	No
P4	0.518	No	O4	1.081	Yes
P5	0.551	No	O5	1.044	No
P6	0.635	No	O6	0.376	No
P7	0.396	No	O7	0.613	No
P8	0.443	No	O8	0.377	No
P9	-0.085	Yes	O9	1.088	No
P10	-0.115	Yes	O10	1.654	No
P11	0.500	No	O11	2.176	No
P12	0.500	No	O12	1.511	No
P13	0.541	No	O13	1.211	No
P14	0.478	No	O14	1.172	No
P15	0.479	No	O15	0.817	No
P16	-0.169	No	O16	1.739	No
P17	0.183	No	O17	0.905	No
P18	0.183	No	O18	0.187	No
P19	-0.369	No	O19	1.282	No
P20	0.407	No	O20	1.641	No
P21	0.477	No	O21	1.720	No
P22	0.508	No	T1	0.640	No

ROCT2: renal organic cation transporter 2.

Table S6. Toxicity of phenolics and essential oils identified in *P. odorata* extracts.

Toxicity						
**Codes of Compounds**	**AMES Test (Yes/No)**	**MRTD (log mg/kg/day)**	**hERG I & II Inhibitors (Yes/No)**	**Oral Rat Acute Toxicity (LD_{50}) (mol/kg)**	**Hepatotoxicity (Yes/No)**	***T. Pyriformis* Toxicity (log μg/L)**
**A. Phenolics**						
P1	No	-0.134	No / No	1.973	No	0.285
P2	No	1.193	No / No	2.240	No	0.262
P3	No	1.145	No / No	2.383	No	0.293
P4	No	0.700	No / No	2.218	No	0.285
P5	No	0.814	No / No	2.423	No	0.273
P6	Yes	-0.296	No / No	1.898	No	0.195
P7	No	0.507	No / No	2.171	No	0.285
P8	No	0.391	No / Yes	2.002	No	0.285
P9	No	0.438	No / Yes	2.482	No	0.285

P10	No	0.438	No / Yes	2.482	No	0.285
P11	No	0.538	No / Yes	2.669	No	0.285
P12	No	0.538	No / Yes	2.669	No	0.285
P13	No	0.534	No / No	2.664	No	0.285
P14	No	0.650	No / Yes	2.879	No	0.285
P15	No	0.651	No / Yes	2.889	No	0.285
P16	No	0.449	No / Yes	2.558	No	0.285
P17	No	0.438	No / No	2.428	No	0.347
P18	No	0.438	No / No	2.428	No	0.347
P19	No	0.452	No / Yes	2.491	No	0.285
P20	No	0.499	No / No	2.471	No	0.288
P21	No	0.531	No / No	2.449	No	0.312
P22	No	0.576	No / No	2.407	No	0.296
T1	No	0.793	No / No	2.357	No	0.285
			**B. Essential Oils**			
O1	No	0.423	No / No	1.577	No	1.238
O2	No	0.273	No / No	1.523	No	1.926
O3	No	0.351	No / No	1.617	No	1.401
O4	No	-0.007	No / No	1.739	No	0.994
O5	No	-0.041	No / No	1.616	No	1.641
O6	No	0.543	No / No	1.815	No	1.036
O7	No	0.294	No / No	2.103	No	0.733
O8	No	0.669	No / No	1.576	No	0.469
O9	No	0.351	No / No	1.617	No	1.401
O10	No	0.389	No / No	1.597	No	1.639
O11	No	-0.222	No / Yes	1.716	No	0.371
O12	No	1.016	No / No	1.873	No	1.869
O13	No	-0.018	No / No	1.543	No	1.623
O14	No	-0.018	No / No	1.543	No	1.623
O15	No	-0.193	No / No	1.615	No	1.369
O16	No	0.253	No / No	1.580	No	2.284
O17	No	0.148	No / No	1.548	No	1.079
O18	No	0.684	No / No	2.042	No	1.119
O19	No	0.551	No / No	1.766	No	1.451
O20	No	0.501	No / No	1.614	No	1.308
O21	No	0.312	No / No	1.573	No	2.100

AMES test (No), MRTD: max recommended tolerated dose showed in log mg/kg/day (low ≤ 0.477, high > 0.477), hERG I and II: human ether-a-go-go gene (No), ORAL-LD_{50}: oral rat acute toxicity-half-lethal dose showed in mol/kg, Hepatotoxicity (No), *T. Piryformis* toxicity showed in (log µg/L (> -0.5 = toxic).
